# Correction: Additive effects on the energy barrier for synaptic vesicle fusion cause supralinear effects on the vesicle fusion rate

**DOI:** 10.7554/eLife.09036

**Published:** 2015-06-04

**Authors:** Sebastiaan Schotten, Marieke Meijer, Alexander Matthias Walter, Vincent Huson, Lauren Mamer, Lawrence Kalogreades, Mirelle ter Veer, Marvin Ruiter, Nils Brose, Christian Rosenmund, Jakob Balslev Sørensen, Matthijs Verhage, Lennart Niels Cornelisse

Schotten S, Meijer M, Walter AM, Huson V, Mamer L, Kalogreades L, ter Veer M, Ruiter M, Brose N, Rosenmund C, Sørensen JB, Verhage M, Cornelisse LN. 2015. Additive effects on the energy barrier for synaptic vesicle fusion cause supralinear effects on the vesicle fusion rate. *eLife* **4**:e05531. doi: 10.7554/eLife.05531

Published 14 April 2015

In the published article, minus signs were missing from the exponents in equations 13 and 14:

(13) $$R\left(t\right)=R_{0}e^{-k_{2,\max}\left(\tau e^{\frac{t}{\tau }}+t\right)+k_{2,\max}\tau },$$

(14) $$\begin{array}{ll} \frac{dF}{dt} & =k_{2}\left(t\right)R\\ & =k_{2,\max}\left(1-e^{-\frac{t}{\tau }}\right)R_{0}e^{-k_{2,\max}\left(\tau e^{\frac{t}{\tau }}+t\right)+k_{2,\max}\tau }. \end{array}$$

These equations should have read:

(13) $$R\left(t\right)=R_{0}e^{-k_{2,\max}\left(\tau e^{-\frac{t}{\tau }}+t\right)+k_{2,\max}\tau },$$

(14) $$\begin{array}{ll} \frac{dF}{dt} & =k_{2}\left(t\right)R\\ & =k_{2,\max}\left(1-e^{-\frac{t}{\tau }}\right)R_{0}e^{-k_{2,\max}\left(\tau e^{-\frac{t}{\tau }}+t\right)+k_{2,\max}\tau }. \end{array}$$

We apologize for any confusion that may have occurred.

The article has been corrected accordingly.

