# Supplementary material for: Additive effects on the energy barrier for synaptic vesicle fusion cause supralinear effects on the vesicle fusion rate
Source: eLife. 2015 Apr 14;4:e05531. doi: 10.7554/eLife.05531 (PMC4426983; doi:10.7554/eLife.05531)
Supplement: Figure 2—source data 1. — DOI: http://dx.doi.org/10.7554/eLife.05531.005 [file elife05531s001.docx]

**Figure 2-source data 1**

| mEPSC amplitude | 15 pA |
| --- | --- |
| mEPSC time constant rise phase | 200 $\mu$s |
| mEPSC decay time constant | 3 ms |
| $\tau$ | 0.5 s |
| $k_{2,max}$ | 0.5, 3, 5, 10 s^-1^ |

**Parameter values Figure 2-figure supplement 1**

| $k_{1}$ | 0.045, 0.09, 0.18 s^-1^ |
| --- | --- |
| $D$ | 500, 1000, 2000 pC |
| $k_{-1}$ | 0.08, 0.16, 0.32 s^-1^ |
| $t_{del}$ | 0.30, 0.60, 1.20 s |
| $k_{2,max}$ | 1.75, 3.5, 7.0 s^-1^ |
| $\tau$ | 0.10, 0.20, 0.40 s |

**Parameter values Figure 2-figure supplement 3**
