# Supplementary material for: Additive effects on the energy barrier for synaptic vesicle fusion cause supralinear effects on the vesicle fusion rate
Source: eLife. 2015 Apr 14;4:e05531. doi: 10.7554/eLife.05531 (PMC4426983; doi:10.7554/eLife.05531)
Supplement: Figure 3—source data 1. — DOI: http://dx.doi.org/10.7554/eLife.05531.010 [file elife05531s002.docx]

**Figure 3-source data 1**

|  | 0.5M | 0.75M | 1M | p-value |
| --- | --- | --- | --- | --- |
| RRP size | 1.31 ± 0.23 nC (n=22) | 1.40 ± 0.27 nC (n=13) | 1.74 ± 0.43 nC (n=18) | > 0.05 (ANOVA) |

**Parameter values Figure 3B**

|  | 0M | 0.25M | 0.5M | 0.75M | 1M | p-value |
| --- | --- | --- | --- | --- | --- | --- |
| $k_{2,max}$ | 4.30 ± 0.82·10^-4^ s^-1^ (n = 12) | 1.93 ± 0.55·10^-1^ s^-1^ (n = 6) | 2.52 ± 0.21 s^-1^ (n=22) | 3.65 ± 0.68 s^-1^ (n = 13) | 4.49 ± 0.39 s^-1^ (n = 18) | < 0.05 for all combinations except 0.75-1.0M and 0.5-0.75M (ANOVA + Bonferroni post-hoc test) |

**Parameter values Figure 3C**

|  | 0M | 0.25M | 0.5M | 0.75M | 1M | p-value |
| --- | --- | --- | --- | --- | --- | --- |
| $E_{a}$ | 0.0 ± 0.2 RT | -6.1 ± 0.3 RT | -8.7 ± 0.1 RT | -9.0 ± 0.2 RT | -9.3 ± 0.1 RT | As in figure C |

| Fitted function (energy barrier domain) | $E_{a}=ae^{-bx}+c$ |
| --- | --- |
| Transformed function (rate constant domain) | $k_{2,max}=k_{2,max}(0M)\cdot e^{-(ae^{-bx}+c)/RT}$ |
|  |  |
| Fitted parameter | Value (unit) |
| a | 17.09 (RT) |
| b | 6.81 (M^-1^) |
| c | -9.22 (RT) |

**Parameter values Figure 3D**

| Parameter | Mean | Std | 95% CI | Unit | Mean ± SEM (fig 4) |
| --- | --- | --- | --- | --- | --- |
| $RRP(0.5M)$ | 1310.1 | 222.0 | [896.3, 1758.2] | pC | 1.31 ± 0.23 nC  (n=22) |
| $RRP(0.75M)$ | 1400.1 | 256.0 | [915.7, 1924.0] | pC | 1.40 ± 0.27 nC  (n=13) |
| $RRP(1M)$ | 1740.0 | 417.6 | [1048.8, 2648.7] | pC | 1.74 ± 0.43 nC  (n=18) |
|  |  |  |  |  |  |
| $k_{2,max}(0M)$ | 4.30·10^-4^ | 0.79·10^-4^ | [2.87, 5.96]·10^-4^ | 1/s | 4.30 ± 0.82·10^-4^ 1/s  (n=12) |
| $k_{2,max}(0.25M)$ | 1.93·10^-1^ | 0.50·10^-1^ | [1.18, 3.04]·10^-1^ | 1/s | 1.93 ± 0.55·10^-1^ 1/s  (n=6) |
| $k_{2,max}(0.5M)$ | 2.52 | 0.21 | [2.12, 2.94] | 1/s | 2.52 ± 0.21 1/s  (n=22) |
| $k_{2,max}(0.75M)$ | 3.66 | 0.66 | [2.56, 5.12] | 1/s | 3.65 ± 0.68 1/s  (n=13) |
| $k_{2,max}(1M)$ | 4.50 | 0.38 | [3.79, 5.24] | 1/s | 4.49 ± 0.39 1/s  (n=18) |

**Parameter values bootstrap analysis Figure 3**

|  | 500mM | 750mM | 1000mM | p-value |
| --- | --- | --- | --- | --- |
| $k_{1}D$ | 132 ± 31 pA (n = 22) | 98 ± 26 pA (n = 13) | 81 ± 15 pA (n = 18) | > 0.05 (ANOVA) |

**Parameter values Figure 3-figure supplement 1A**

|  | 500mM | 750mM | 1000mM | p-value |
| --- | --- | --- | --- | --- |
| $k_{-1}$ | 0.11 ± 0.01 s^-1^ (n = 22) | 0.09 ± 0.02s^-1^ pA (n = 13) | 0.07 ± 0.02 s^-1^ (n = 18) | > 0.05 (ANOVA) |

**Parameter values Figure 3-figure supplement 1B**

|  | 250mM | 500mM | 750mM | 1000mM | p-value |
| --- | --- | --- | --- | --- | --- |
| $t_{del}$ | 1.58 ± 0.32 s (n = 6) | 1.24 ± 0.07 s (n = 22) | 1.08 ± 0.03 s (n = 13) | 1.07 ± 0.04 s (n = 18) | > 0.05 for all combinations except 0.25-0.75M and 0.25-1M (ANOVA + Bonferroni post-hoc test) |

**Parameter values Figure 3-figure supplement 1C**

| Parameter | Mean | Std | 95% CI | Unit | Mean ± SEM (fig4S1) |
| --- | --- | --- | --- | --- | --- |
| $k_{1}D(0.5M)$ | 132 | 30 | [80, 196] | pA | 132 ± 31 pA  (n=22) |
| $k_{1}D(0.75M)$ | 98 | 25 | [54, 151] | pA | 98 ± 26 pA  (n=13) |
| $k_{1}D(1M)$ | 81 | 14 | [55, 109] | pA | 81 ± 15 pA  (n=18) |
|  |  |  |  |  |  |
| $k_{-1}(0.5M)$ | 1.1·10^-1^ | 0.1·10^-1^ | [0.8, 1.4]·10^-1^ | 1/s | 1.1 ± 0.1·10^-1^ 1/s  (n=22) |
| $k_{-1}(0.75M)$ | 0.9·10^-1^ | 0.2·10^-1^ | [0.5, 1.4]·10^-1^ | 1/s | 0.9 ± 0.2·10^-1^ 1/s  (n=13) |
| $k_{-1}(1M)$ | 0.7·10^-1^ | 0.2·10^-1^ | [0.4, 1.1]·10^-1^ | 1/s | 0.7 ± 0.2·10^-1^ 1/s  (n=18) |
|  |  |  |  |  |  |
| $t_{del}(0.25M)$ | 1.58 | 0.29 | [1.19, 2.22] | s | 1.58 ± 0.32 s (n = 6) |
| $t_{del}(0.5M)$ | 1.24 | 0.07 | [1.11, 1.37] | s | 1.24 ± 0.07 s (n = 22) |
| $t_{del}(0.75M)$ | 1.08 | 0.03 | [1.03, 1.14] | s | 1.08 ± 0.03 s (n = 13) |
| $t_{del}(1M)$ | 1.07 | 0.04 | [0.99, 1.16] | s | 1.07 ± 0.04 s (n = 18) |

**Parameter values bootstrap analysis Figure 3-figure supplement 1**

|  | +KYN/-KYN | p-value |
| --- | --- | --- |
| $k_{2,max}(0.5M)$ | 1.08 ± 0.04 (n = 9) | 8.2·10^-2^ (Paired t-test) |
| $k_{2,max}(0.75M)$ | 1.28 ± 0.08 (n = 5) | 2.2·10^-2^ (Paired t-test) |

**Parameter values Figure 3-figure supplement 3B**

|  | +KYN/-KYN | p-value |
| --- | --- | --- |
| $RRP(0.5M)$ | 0.52 ± 0.01 (n = 9) | 1.0·10^-3^ (Paired t-test) |
| $RRP(0.75M)$ | 0.63 ± 0.02 (n = 5) | 2.3·10^-2^ (Paired t-test) |

**Parameter values Figure 3-figure supplement 3C**

|  | +KYN/-KYN | p-value |
| --- | --- | --- |
| $k_{1}D(0.5M)$ | 0.40 ± 0.06 (n = 9) | 4.9·10^-3^ (Paired t-test) |
| $k_{1}D(0.75M)$ | 0.31 ± 0.07 (n = 5) | 0.8·10^-3^ (Paired t-test) |

**Parameter values Figure 3-figure supplement 3D**

|  | +KYN/-KYN | p-value |
| --- | --- | --- |
| $k_{-1}(0.5M)$ | 0.75 ± 0.11 (n = 9) | 5.7·10^-2^ (Paired t-test) |
| $k_{-1}(0.75M)$ | 0.48 ± 0.08 (n = 5) | 3.5·10^-3^ (Paired t-test) |

**Parameter values Figure 3-figure supplement 3E**

|  | 0.5M | Corrected | p-value |
| --- | --- | --- | --- |
| $k_{1}D$ | 265 ± 80 pA (n = 7) | 293 ± 91 pA (n = 7) | > 0.05 (Paired t-test) |

**Parameter values Figure 3-figure supplement 4B**

|  | 0.5M | Corrected | p-value |
| --- | --- | --- | --- |
| $k_{-1}$ | 0.11 ± 0.01 s^-1^ (n = 7) | 0.12 ± 0.01 s^-1^ (n = 7) | > 0.05 (Paired t-test) |

**Parameter values Figure 3-figure supplement 4C**

|  | 0.5M | Corrected | p-value |
| --- | --- | --- | --- |
| $k_{2,max}$ | 2.02 ± 0.16 s^-1^ (n = 7) | 1.92 ± 0.20 s^-1^ (n = 7) | > 0.05 (Paired t-test) |

**Parameter values Figure 3-figure supplement 4D**

|  | 0.5M | Corrected | p-value |
| --- | --- | --- | --- |
| $RRP$ | 2.43 ± 0.65 nC (n = 7) | 2.44 ± 0.64 nC (n = 7) | > 0.05 (Paired t-test) |

**Parameter values Figure 3-figure supplement 4E**
