# Supplementary material for: Additive effects on the energy barrier for synaptic vesicle fusion cause supralinear effects on the vesicle fusion rate
Source: eLife. 2015 Apr 14;4:e05531. doi: 10.7554/eLife.05531 (PMC4426983; doi:10.7554/eLife.05531)
Supplement: Figure 3—source data 2. — DOI: http://dx.doi.org/10.7554/eLife.05531.011 [file elife05531s003.docx]

**Figure 3-source data 2**

All traces in Figure3-figure supplement 5A,B were simulated using the default parameters [k1, k-1, k2max, tdel, τ] = [0.09, 0.16, 3.50, 0.60, 0.20], unless indicated otherwise, with superimposed Gaussian white noise (µ=0, σ=10pA).

| Trace | Fit | K1 | K-1 | K2max | tdel | Τ | D | R | cost |
| --- | --- | --- | --- | --- | --- | --- | --- | --- | --- |
| #1 |  | **0.09** | **0.16** | **3.50** | **0.60** | **0.20** | **1000** | **562.5** |  |
|  | #1 | 0.0901 | 0.1603 | 3.5044 | 0.6003 | 0.1999 | 1000 | 561.8498 | 6.8502e+005 |
|  | #2 | 0.0901 | 0.1603 | 3.5044 | 0.6003 | 0.1999 | 1000 | 561.8498 | 6.8502e+005 |
|  | #3 | 0.0866 | 0.1482 | 2.4909 | 0.5486 | 0.1207 | 1000 | 583.9361 | 2.7243e+006 |
|  | #4 | 0.0841 | 0.1325 | 2.0908 | 0.5233 | 0.1044 | 1000 | 635.0144 | 4.3122e+006 |
|  | #5 | 0.0901 | 0.1603 | 3.5044 | 0.6003 | 0.1999 | 1000 | 561.8498 | 6.8502e+005 |
|  | #6 | 0.0860 | 0.1470 | 2.8025 | 0.5619 | 0.1591 | 1000 | 585.0824 | 1.1871e+006 |
|  | #7 | 0.0901 | 0.1603 | 3.5044 | 0.6003 | 0.1999 | 1000 | 561.8498 | 6.8502e+005 |
|  | #8 | 0.0856 | 0.1441 | 2.8639 | 0.5618 | 0.1665 | 1000 | 594.2993 | 1.2445e+006 |
|  | #9 | 0.0901 | 0.1603 | 3.5044 | 0.6003 | 0.1999 | 1000 | 561.8498 | 6.8502e+005 |
|  | #10 | 0.0901 | 0.1603 | 3.5044 | 0.6003 | 0.1999 | 1000 | 561.8498 | 6.8502e+005 |

**Parameter values for Figure 3-figure supplement 5A1:** Rejected upon visual inspection: #3, 4.

| Trace | Fit | K1 | K-1 | K2max | tdel | Τ | D | R | cost |
| --- | --- | --- | --- | --- | --- | --- | --- | --- | --- |
| #1 |  | **0.045** | **0.16** | **3.50** | **0.60** | **0.20** | **1000** | **281.25** |  |
|  | #1 | 0.0450 | 0.1604 | 3.5145 | 0.6009 | 0.2003 | 1000 | 280.5762 | 6.9684e+005 |
|  | #2 | 0.0156 | 0.0313 | 0.6774 | 0.3950 | 0.1459 | 1000 | 499.6188 | 1.5877e+007 |
|  | #3 | 0.0364 | 0.1072 | 2.0022 | 0.5214 | 0.1145 | 1000 | 339.7266 | 1.8571e+006 |
|  | #4 | 0.0450 | 0.1604 | 3.5145 | 0.6009 | 0.2003 | 1000 | 280.5762 | 6.9684e+005 |
|  | #5 | 0.0206 | 0.0444 | 0.8399 | 0.4602 | 0.0627 | 1000 | 463.3061 | 1.0279e+007 |
|  | #6 | 0.0411 | 0.1279 | 2.0637 | 0.5286 | 0.1115 | 1000 | 321.4415 | 1.5826e+006 |
|  | #7 | 0.0450 | 0.1604 | 3.5145 | 0.6009 | 0.2003 | 1000 | 280.5762 | 6.9684e+005 |
|  | #8 | 0.0411 | 0.1279 | 2.0637 | 0.5286 | 0.1115 | 1000 | 321.4415 | 1.5826e+006 |
|  | #9 | 0.0450 | 0.1604 | 3.5145 | 0.6009 | 0.2003 | 1000 | 280.5762 | 6.9684e+005 |
|  | #10 | 0.0450 | 0.1604 | 3.5145 | 0.6009 | 0.2003 | 1000 | 280.5762 | 6.9684e+005 |

**Parameter values for Figure 3-figure supplement 5A2 left trace:** k1 = 0.09 decreased to k1 = 0.045. Rejected upon visual inspection: #2, 3, 5, 6, 8.

| Trace | Fit | K1 | K-1 | K2max | tdel | Τ | D | R | cost |
| --- | --- | --- | --- | --- | --- | --- | --- | --- | --- |
| #1 |  | **0.18** | **0.16** | **3.50** | **0.60** | **0.20** | **1000** | **1125.0** |  |
|  | #1 | 0.1738 | 0.1381 | 2.1114 | 0.5288 | 0.1020 | 1000 | 1258.3 | 1.5458e+007 |
|  | #2 | 0.1799 | 0.1600 | 3.5081 | 0.6006 | 0.2005 | 1000 | 1124.5 | 6.8146e+005 |
|  | #3 | 0.1799 | 0.1600 | 3.5081 | 0.6006 | 0.2005 | 1000 | 1124.5 | 6.8146e+005 |
|  | #4 | 0.1799 | 0.1600 | 3.5081 | 0.6006 | 0.2005 | 1000 | 1124.5 | 6.8146e+005 |
|  | #5 | 0.1799 | 0.1600 | 3.5081 | 0.6006 | 0.2005 | 1000 | 1124.5 | 6.8146e+005 |
|  | #6 | 0.1642 | 0.1273 | 2.0593 | 0.5280 | 0.1122 | 1000 | 1289.4 | 1.4853e+007 |
|  | #7 | 0.1799 | 0.1600 | 3.5081 | 0.6006 | 0.2005 | 1000 | 1124.5 | 6.8146e+005 |
|  | #8 | 0.0034 | 0.0023 | 1.2665 | 0.5292 | 0.0378 | 1000 | 1490.5 | 1.6299e+008 |
|  | #9 | 0.1799 | 0.1600 | 3.5081 | 0.6006 | 0.2005 | 1000 | 1124.5 | 6.8146e+005 |
|  | #10 | 0.1316 | 0.0992 | 1.7397 | 0.5248 | 0.0730 | 1000 | 1327.1 | 4.0086e+007 |

**Parameter values for Figure 3-figure supplement 5A2 right trace:** k1 = 0.09 increased to k1 = 0.18. Rejected upon visual inspection: #1, 6, 8, 10.

| Trace | Fit | K1 | K-1 | K2max | tdel | Τ | D | R | cost |
| --- | --- | --- | --- | --- | --- | --- | --- | --- | --- |
| #1 |  | **0.09** | **0.08** | **3.50** | **0.60** | **0.20** | **1000** | **1125.0** |  |
|  | #1 | 0.0747 | 0.0598 | 2.1442 | 0.5252 | 0.1041 | 1000 | 1249.7 | 1.6044e+007 |
|  | #2 | 0.0899 | 0.0800 | 3.4996 | 0.6002 | 0.1997 | 1000 | 1124.5 | 7.0826e+005 |
|  | #3 | 0.0719 | 0.0568 | 2.1166 | 0.5297 | 0.1106 | 1000 | 1264.1 | 1.5712e+007 |
|  | #4 | 0.0899 | 0.0800 | 3.4996 | 0.6002 | 0.1997 | 1000 | 1124.5 | 7.0826e+005 |
|  | #5 | 0.0740 | 0.0588 | 2.1246 | 0.5236 | 0.1070 | 1000 | 1258.1 | 1.5890e+007 |
|  | #6 | 0.0899 | 0.0800 | 3.4996 | 0.6002 | 0.1997 | 1000 | 1124.5 | 7.0826e+005 |
|  | #7 | 0.0899 | 0.0800 | 3.4996 | 0.6002 | 0.1997 | 1000 | 1124.5 | 7.0826e+005 |
|  | #8 | 0.0820 | 0.0661 | 2.1352 | 0.5254 | 0.1050 | 1000 | 1239.4 | 1.6284e+007 |
|  | #9 | 0.0893 | 0.0790 | 3.4481 | 0.5971 | 0.1999 | 1000 | 1129.8 | 7.4627e+005 |
|  | #10 | 0.0899 | 0.0800 | 3.4996 | 0.6002 | 0.1997 | 1000 | 1124.5 | 7.0826e+005 |

**Parameter values for Figure 3-figure supplement 5A3 left trace:** k-1 = 0.16 decreased to k-1 = 0.08. Rejected upon visual inspection: #1, 3, 5, 8.

| Trace | Fit | K1 | K-1 | K2max | tdel | Τ | D | R | cost |
| --- | --- | --- | --- | --- | --- | --- | --- | --- | --- |
| #1 |  | **0.09** | **0.32** | **3.50** | **0.60** | **0.20** | **1000** | **281.25** |  |
|  | #1 | 0.0898 | 0.3189 | 3.4961 | 0.6003 | 0.1997 | 1000 | 281.7811 | 6.8631e+005 |
|  | #2 | 0.0898 | 0.3189 | 3.4961 | 0.6003 | 0.1997 | 1000 | 281.7811 | 6.8631e+005 |
|  | #3 | 0.0903 | 0.2843 | 2.0962 | 0.5320 | 0.1273 | 1000 | 317.6228 | 1.2558e+006 |
|  | #4 | 0.0891 | 0.2660 | 1.9570 | 0.5266 | 0.1143 | 1000 | 334.8679 | 1.4459e+006 |
|  | #5 | 0.0898 | 0.3189 | 3.4961 | 0.6003 | 0.1997 | 1000 | 281.7811 | 6.8631e+005 |
|  | #6 | 0.0890 | 0.2661 | 1.9617 | 0.5221 | 0.1118 | 1000 | 334.3981 | 1.4422e+006 |
|  | #7 | 0.0887 | 0.1721 | 0.6164 | 0.0053 | 0.3725 | 1000 | 515.5299 | 2.7636e+007 |
|  | #8 | 0.0898 | 0.3189 | 3.4961 | 0.6003 | 0.1997 | 1000 | 281.7811 | 6.8631e+005 |
|  | #9 | 0.0890 | 0.2655 | 1.9599 | 0.5266 | 0.1137 | 1000 | 335.1188 | 1.4453e+006 |
|  | #10 | 0.0898 | 0.3189 | 3.4961 | 0.6003 | 0.1997 | 1000 | 281.7811 | 6.8631e+005 |

**Parameter values for Figure 3-figure supplement 5A3 right trace:** k-1 = 0.16 increased to k-1 = 0.32. Rejected upon visual inspection: #3, 4, 6, 7, 9.

| Trace | Fit | K1 | K-1 | K2max | tdel | Τ | D | R | cost |
| --- | --- | --- | --- | --- | --- | --- | --- | --- | --- |
| #1 |  | **0.09** | **0.16** | **1.75** | **0.60** | **0.20** | **1000** | **562.5** |  |
|  | #1 | 0.2064 | 0.7966 | 1.9642 | 0.5818 | 0.0649 | 1000 | 259.1592 | 4.7865e+007 |
|  | #2 | 0.0894 | 0.1594 | 1.7542 | 0.5996 | 0.2029 | 1000 | 561.1647 | 7.0778e+005 |
|  | #3 | 0.0895 | 0.1596 | 1.7498 | 0.5990 | 0.2007 | 1000 | 560.8608 | 7.0674e+005 |
|  | #4 | 0.0895 | 0.1596 | 1.7498 | 0.5990 | 0.2007 | 1000 | 560.8608 | 7.0674e+005 |
|  | #5 | 0.0835 | 0.1352 | 1.1805 | 0.5559 | 0.0924 | 1000 | 617.6704 | 3.3845e+006 |
|  | #6 | 0.0895 | 0.1596 | 1.7498 | 0.5990 | 0.2007 | 1000 | 560.8608 | 7.0674e+005 |
|  | #7 | 0.0887 | 0.1560 | 1.6698 | 0.5916 | 0.1907 | 1000 | 568.4378 | 7.2290e+005 |
|  | #8 | 0.0895 | 0.1596 | 1.7498 | 0.5990 | 0.2007 | 1000 | 560.8608 | 7.0674e+005 |
|  | #9 | 0.0895 | 0.1596 | 1.7498 | 0.5990 | 0.2007 | 1000 | 560.8608 | 7.0674e+005 |
|  | #10 | 0.0895 | 0.1596 | 1.7498 | 0.5990 | 0.2007 | 1000 | 560.8608 | 7.0674e+005 |

**Parameter values for Figure 3-figure supplement 5A4 left trace:** k2max = 3.50 decreased to k2max = 1.75. Rejected upon visual inspection: #1, 5.

| Trace | Fit | K1 | K-1 | K2max | tdel | Τ | D | R | cost |
| --- | --- | --- | --- | --- | --- | --- | --- | --- | --- |
| #1 |  | **0.09** | **0.16** | **7.00** | **0.60** | **0.20** | **1000** | **562.5** |  |
|  | #1 | 0.0782 | 0.1208 | 2.9351 | 0.4863 | 0.0887 | 1000 | 647.5144 | 1.1525e+007 |
|  | #2 | 0.0896 | 0.1590 | 7.0076 | 0.6007 | 0.2008 | 1000 | 563.4154 | 7.0090e+005 |
|  | #3 | 0.0896 | 0.1590 | 7.0076 | 0.6007 | 0.2008 | 1000 | 563.4154 | 7.0090e+005 |
|  | #4 | 0.0896 | 0.1590 | 7.0076 | 0.6007 | 0.2008 | 1000 | 563.4154 | 7.0090e+005 |
|  | #5 | 0.0751 | 0.1021 | 2.4329 | 0.4826 | 0.0605 | 1000 | 735.4188 | 2.1495e+007 |
|  | #6 | 0.0750 | 0.1018 | 2.4279 | 0.4821 | 0.0608 | 1000 | 736.6161 | 2.1571e+007 |
|  | #7 | 0.0896 | 0.1590 | 7.0076 | 0.6007 | 0.2008 | 1000 | 563.4154 | 7.0090e+005 |
|  | #8 | 0.0896 | 0.1590 | 7.0076 | 0.6007 | 0.2008 | 1000 | 563.4154 | 7.0090e+005 |
|  | #9 | 0.0896 | 0.1590 | 7.0076 | 0.6007 | 0.2008 | 1000 | 563.4154 | 7.0090e+005 |
|  | #10 | 0.0896 | 0.1590 | 7.0076 | 0.6007 | 0.2008 | 1000 | 563.4154 | 7.0090e+005 |

**Parameter values for Figure 3-figure supplement 5A4 right trace:** k2max = 3.50 increased to k2max = 7.00: Rejected upon visual inspection: #1, 5, 6.

| Trace | Fit | K1 | K-1 | K2max | tdel | Τ | D | R | cost |
| --- | --- | --- | --- | --- | --- | --- | --- | --- | --- |
| #1 |  | **0.09** | **0.16** | **3.50** | **0.30** | **0.20** | **1000** | **562.5** |  |
|  | #1 | 0.0904 | 0.1607 | 3.5070 | 0.3005 | 0.2006 | 1000 | 562.4766 | 7.0952e+005 |
|  | #2 | 0.0763 | 0.1060 | 1.3941 | 0.0617 | 0.1736 | 1000 | 719.2001 | 3.6057e+007 |
|  | #3 | 0.0876 | 0.1518 | 2.8822 | 0.2710 | 0.1560 | 1000 | 577.5091 | 1.2799e+006 |
|  | #4 | 0.0904 | 0.1607 | 3.5070 | 0.3005 | 0.2006 | 1000 | 562.4766 | 7.0952e+005 |
|  | #5 | 0.0904 | 0.1607 | 3.5070 | 0.3005 | 0.2006 | 1000 | 562.4766 | 7.0952e+005 |
|  | #6 | 0.0841 | 0.1296 | 1.7958 | 0.1752 | 0.1148 | 1000 | 648.5920 | 1.0059e+007 |
|  | #7 | 0.0869 | 0.1697 | 1.9184 | 0.2626 | 0.0379 | 1000 | 512.3039 | 1.8366e+007 |
|  | #8 | 0.0904 | 0.1607 | 3.5070 | 0.3005 | 0.2006 | 1000 | 562.4766 | 7.0952e+005 |
|  | #9 | 0.0904 | 0.1607 | 3.5070 | 0.3005 | 0.2006 | 1000 | 562.4766 | 7.0952e+005 |
|  | #10 | 0.0904 | 0.1607 | 3.5070 | 0.3005 | 0.2006 | 1000 | 562.4766 | 7.0952e+005 |

**Parameter values for Figure 3-figure supplement 5A5 left trace:** tdel = 0.60 decreased to tdel = 0.30: Rejected upon visual inspection: #2, 6, 7.

| Trace | Fit | K1 | K-1 | K2max | tdel | Τ | D | R | cost |
| --- | --- | --- | --- | --- | --- | --- | --- | --- | --- |
| #1 |  | **0.09** | **0.16** | **3.50** | **1.20** | **0.20** | **1000** | **562.5** |  |
|  | #1 | 0.0901 | 0.1587 | 3.4827 | 1.1983 | 0.1976 | 1000 | 567.5274 | 7.1475e+005 |
|  | #2 | 0.0871 | 0.1479 | 2.6239 | 1.1551 | 0.1424 | 1000 | 588.8923 | 1.7450e+006 |
|  | #3 | 0.0901 | 0.1587 | 3.4827 | 1.1983 | 0.1976 | 1000 | 567.5274 | 7.1475e+005 |
|  | #4 | 0.0901 | 0.1587 | 3.4827 | 1.1983 | 0.1976 | 1000 | 567.5274 | 7.1475e+005 |
|  | #5 | 0.0901 | 0.1598 | 3.4833 | 1.1988 | 0.1963 | 1000 | 563.8752 | 7.1920e+005 |
|  | #6 | 0.1222 | 0.2454 | 2.1652 | 1.1977 | 0.0412 | 1000 | 498.2355 | 2.9570e+007 |
|  | #7 | 0.0736 | 0.1128 | 2.0372 | 1.1191 | 0.1303 | 1000 | 652.1806 | 4.6592e+006 |
|  | #8 | 0.0888 | 0.1519 | 3.0227 | 1.1753 | 0.1778 | 1000 | 584.8873 | 9.0421e+005 |
|  | #9 | 0.0901 | 0.1587 | 3.4827 | 1.1983 | 0.1976 | 1000 | 567.5274 | 7.1475e+005 |
|  | #10 | 0.0904 | 0.1581 | 3.4526 | 1.2015 | 0.1997 | 1000 | 572.0935 | 7.6703e+005 |

**Parameter values for Figure 3-figure supplement 5A5 right trace:** tdel = 0.60 increased to tdel = 1.20. Rejected upon visual inspection: #6, 7.

| Trace | Fit | K1 | K-1 | K2max | tdel | Τ | D | R | Cost |
| --- | --- | --- | --- | --- | --- | --- | --- | --- | --- |
| #1 |  | **0.09** | **0.16** | **3.50** | **0.60** | **0.10** | **1000** | **562.5** |  |
|  | #1 | 0.0842 | 0.1371 | 2.6632 | 0.5782 | 0.0687 | 1000 | 614.3451 | 2.6979e+006 |
|  | #2 | 0.0833 | 0.1429 | 2.3610 | 0.5785 | 0.0432 | 1000 | 582.7620 | 7.5594e+006 |
|  | #3 | 0.0897 | 0.1593 | 3.5132 | 0.6006 | 0.1005 | 1000 | 562.7842 | 6.9230e+005 |
|  | #4 | 0.0872 | 0.1510 | 3.1855 | 0.5928 | 0.0892 | 1000 | 577.2884 | 9.1266e+005 |
|  | #5 | 0.0897 | 0.1593 | 3.5132 | 0.6006 | 0.1005 | 1000 | 562.7842 | 6.9230e+005 |
|  | #6 | 0.0832 | 0.1363 | 2.4681 | 0.5776 | 0.0553 | 1000 | 610.1948 | 4.8826e+006 |
|  | #7 | 0.0897 | 0.1593 | 3.5132 | 0.6006 | 0.1005 | 1000 | 562.7842 | 6.9230e+005 |
|  | #8 | 0.0897 | 0.1593 | 3.5132 | 0.6006 | 0.1005 | 1000 | 562.7842 | 6.9230e+005 |
|  | #9 | 0.0845 | 0.1486 | 2.5935 | 0.5745 | 0.0630 | 1000 | 568.5611 | 2.8878e+006 |
|  | #10 | 0.0846 | 0.1488 | 2.5954 | 0.5746 | 0.0629 | 1000 | 568.0738 | 2.8876e+006 |

**Parameter values for Figure 3-figure supplement 5A6 left trace:** Τ = 0.20 decreased to Τ = 0.10. Rejected upon visual inspection: # 2, 4, 6, 9, 10.

| Trace | Fit | K1 | K-1 | K2max | tdel | Τ | D | R | Cost |
| --- | --- | --- | --- | --- | --- | --- | --- | --- | --- |
| #1 |  | **0.09** | **0.16** | **3.50** | **0.60** | **0.40** | **1000** | **562.5** |  |
|  | #1 | 0.0899 | 0.1593 | 3.4596 | 0.5967 | 0.3978 | 1000 | 564.6395 | 7.0657e+005 |
|  | #2 | 0.0899 | 0.1593 | 3.4597 | 0.5966 | 0.3978 | 1000 | 564.6601 | 7.0657e+005 |
|  | #3 | 0.0830 | 0.1181 | 1.4159 | 0.2899 | 0.3321 | 1000 | 703.0947 | 1.3236e+007 |
|  | #4 | 0.0844 | 0.1345 | 1.7082 | 0.4054 | 0.1936 | 1000 | 627.8548 | 3.6598e+006 |
|  | #5 | 0.0900 | 0.1594 | 3.4954 | 0.6007 | 0.4010 | 1000 | 564.2509 | 7.0680e+005 |
|  | #6 | 0.0900 | 0.1594 | 3.4954 | 0.6007 | 0.4010 | 1000 | 564.2509 | 7.0680e+005 |
|  | #7 | 0.0899 | 0.1593 | 3.4596 | 0.5967 | 0.3978 | 1000 | 564.6395 | 7.0657e+005 |
|  | #8 | 0.0900 | 0.1594 | 3.4954 | 0.6007 | 0.4010 | 1000 | 564.2509 | 7.0680e+005 |
|  | #9 | 0.0899 | 0.1712 | 13.8168 | 1.3080 | 0.8394 | 1000 | 525.0659 | 2.3562e+006 |
|  | #10 | 0.0899 | 0.1593 | 3.4597 | 0.5967 | 0.3978 | 1000 | 564.6110 | 7.0657e+005 |

**Parameter values for Figure 3-figure supplement 5A6 right trace:** Τ = 0.20 increased to Τ = 0.40. Rejected upon visual inspection: #3, 4, 9.

|  | K1 | k-1 | K2max | tdel | Τ |
| --- | --- | --- | --- | --- | --- |
| 0.5k1 | $0.5000\pm0.0000$  $(n=5)$ | $1.0025\pm0.0000$  $(n=5)$ | $1.0041\pm0.0000$  $(n=5)$ | $1.0015\pm0.0000$  $(n=5)$ | $1.0015\pm0.0000$  $(n=5)$ |
| k1 | $0.9892\pm0.0078$  $(n=8)$ | $0.9788\pm0.0152$  $(n=8)$ | $0.9533\pm0.0314$  $(n=8)$ | $0.9845\pm0.0105$  $(n=8)$ | $0.9531\pm0.0306$  $(n=8)$ |
| 2k1 | $1.9989\pm0.0000$  $(n=6)$ | $1.0000\pm0.0000$  $(n=6)$ | $1.0023\pm0.0000$  $(n=6)$ | $1.0010\pm0.0000$  $(n=6)$ | $1.0025\pm0.0000$  $(n=6)$ |
| 0.5k-1 | $0.9978\pm0.0011$  $(n=6)$ | $0.4990\pm0.0010$  $(n=6)$ | $0.9974\pm0.0025$  $(n=6)$ | $0.9995\pm0.0009$  $(n=6)$ | $0.9987\pm0.0002$  $(n=6)$ |
| k-1 | $0.9892\pm0.0078$  $(n=8)$ | $0.9788\pm0.0152$  $(n=8)$ | $0.9533\pm0.0314$  $(n=8)$ | $0.9845\pm0.0105$  $(n=8)$ | $0.9531\pm0.0306$  $(n=8)$ |
| 2k-1 | $0.9978\pm0.0000$  $(n=5)$ | $1.9931\pm0.0000$  $(n=5)$ | $0.9989\pm0.0000$  $(n=5)$ | $1.0005\pm0.0000$  $(n=5)$ | $0.9985\pm0.0000$  $(n=5)$ |
| 0.5k2max | $0.9932\pm0.0011$  $(n=8)$ | $0.9945\pm0.0028$  $(n=8)$ | $0.4972\pm0.0029$  $(n=8)$ | $0.9969\pm0.0016$  $(n=8)$ | $0.9986\pm0.0066$  $(n=8)$ |
| K2max | $0.9892\pm0.0078$  $(n=8)$ | $0.9788\pm0.0152$  $(n=8)$ | $0.9533\pm0.0314$  $(n=8)$ | $0.9845\pm0.0105$  $(n=8)$ | $0.9531\pm0.0306$  $(n=8)$ |
| 2k2max | $0.9956\pm0.0000$  $(n=7)$ | $0.9938\pm0.0000$  $(n=7)$ | $2.0022\pm0.0000$  $(n=7)$ | $1.0012\pm0.0000$  $(n=7)$ | $1.0040\pm0.0000$  $(n=7)$ |
| 0.5tdel | $1.0000\pm0.0044$  $(n=7)$ | $0.9964\pm0.0079$  $(n=7)$ | $0.9764\pm0.0255$  $(n=7)$ | $0.4938\pm0.0070$  $(n=7)$ | $0.9711\pm0.0319$  $(n=7)$ |
| Tdel | $0.9892\pm0.0078$  $(n=8)$ | $0.9788\pm0.0152$  $(n=8)$ | $0.9533\pm0.0314$  $(n=8)$ | $0.9845\pm0.0105$  $(n=8)$ | $0.9531\pm0.0306$  $(n=8)$ |
| 2tdel | $0.9956\pm0.0044$  $(n=8)$ | $0.9785\pm0.0094$  $(n=8)$ | $0.9469\pm0.0325$  $(n=8)$ | $1.9841\pm0.0098$  $(n=8)$ | $0.9416\pm0.0351$  $(n=8)$ |
| 0.5τ | $0.9844\pm0.0122$  $(n=5)$ | $0.9679\pm0.0277$  $(n=5)$ | $0.9552\pm0.0486$  $(n=5)$ | $0.9935\pm0.0075$  $(n=5)$ | $0.4707\pm0.0318$  $(n=5)$ |
| τ | $0.9892\pm0.0078$  $(n=8)$ | $0.9788\pm0.0152$  $(n=8)$ | $0.9533\pm0.0314$  $(n=8)$ | $0.9845\pm0.0105$  $(n=8)$ | $0.9531\pm0.0306$  $(n=8)$ |
| 2τ | $0.9994\pm0.0002$  $(n=7)$ | $0.9959\pm0.0001$  $(n=7)$ | $0.9928\pm0.0021$  $(n=7)$ | $0.9973\pm0.0014$  $(n=7)$ | $1.9959\pm0.0032$  $(n=7)$ |

**Data = mean ± SEM (normalised to default parameter value)**

**Parameter values Figure 3-figure supplement 5C**
