# Supplementary material for: Additive effects on the energy barrier for synaptic vesicle fusion cause supralinear effects on the vesicle fusion rate
Source: eLife. 2015 Apr 14;4:e05531. doi: 10.7554/eLife.05531 (PMC4426983; doi:10.7554/eLife.05531)
Supplement: Figure 4—source data 1. — DOI: http://dx.doi.org/10.7554/eLife.05531.018 [file elife05531s004.docx]

**Figure 4-source data 1**

| Model parameter | Value (unit) |
| --- | --- |
| $k_{1}$ | 0.09 (s^-1^) |
| $k_{-1}$ | 0.15 (s^-1^) |
| $D$ | 7.0 (nC) |
| $R$ | 4.4 (nC) |
|  |  |
| Sucrose function parameter |  |
| $k_{2,max}$ | 0.001-20 (s^-1^) |
| $t_{del}$ | 0.36 (s) |
| $\tau$ | 0.20 (s) |
| Duration of sucrose pulse | 7 (s) |

**Parameter values Figure 4B**

| Model parameter | Value (unit) |
| --- | --- |
| $k_{1}$ | 0.09 (s^-1^) |
| $k_{-1}$ | 0.15 (s^-1^) |
| $D$ | 7.0 (nC) |
| $R$ | 4.4 (nC) |
|  |  |
| Sucrose function parameter |  |
| $k_{2,max}$ | 0.01-10 (s^-1^) |
| $t_{del}$ | 0.36 (s) |
| $\tau$ | 0.20 (s) |
| Duration of sucrose pulse | 7 or 20 (s) |

**Parameter values Figure 4-figure supplement 1**
