# Supplementary material for: Additive effects on the energy barrier for synaptic vesicle fusion cause supralinear effects on the vesicle fusion rate
Source: eLife. 2015 Apr 14;4:e05531. doi: 10.7554/eLife.05531 (PMC4426983; doi:10.7554/eLife.05531)
Supplement: Figure 5—source data 1. — DOI: http://dx.doi.org/10.7554/eLife.05531.021 [file elife05531s005.docx]

**Figure 5-source data 1**

| Model parameter | Value (unit) |
| --- | --- |
| $k_{1}$ | 0.09 (s^-1^) |
| $k_{-1}$ | 0.16 (s^-1^) |
| $D$ | 3.0 (nC) |
| $R$ | 1.6 (nC) |
|  |  |
| Sucrose function parameter |  |
| $k_{2,max}$ | 0-30 (s^-1^) |
| $t_{del}$ | 0.60 (s) |
| $\tau$ | 0.20 (s) |
| Duration of sucrose pulse | 7 (s) |

**Parameter values Figure 5**
