# Supplementary material for: Additive effects on the energy barrier for synaptic vesicle fusion cause supralinear effects on the vesicle fusion rate
Source: eLife. 2015 Apr 14;4:e05531. doi: 10.7554/eLife.05531 (PMC4426983; doi:10.7554/eLife.05531)
Supplement: Figure 6—source data 1. — DOI: http://dx.doi.org/10.7554/eLife.05531.023 [file elife05531s006.docx]

**Figure 6-source data 1**

|  | -PDBu | +PDBu | p-value |
| --- | --- | --- | --- |
| $k_{2,max}(0M)$ | 1.70 ± 0.36·10^-3^ s^-1^ (n = 19) | 3.40 ± 0.76·10^-3^ s^-1^ (n = 19) | 9.9·10^-4^ (Paired t-test) |
| $k_{2,max}(0.2M)$ | 1.77 ± 0.50·10^-1^ s^-1^ (n = 20) | 2.49 ± 0.69·10^-1^ s^-1^ (n = 20) | 1.2·10^-2^ (Sign test) |
| $k_{2,max}(0.3M)$ | 5.31 ± 0.84·10^-1^ s^-1^ (n = 18) | 6.81 ± 1.11·10^-1^ s^-1^ (n = 18) | 3.0·10^-2^ (Paired t-test) |
| $k_{2,max}(0.4M)$ | 1.91 ± 0.18 s^-1^ (n = 22) | 2.29 ± 0.20 s^-1^ (n = 22) | 1.5·10^-2^ (Paired t-test) |
| $k_{2,max}(0.5M)$ | 2.29 ± 0.13 s^-1^ (n = 36) | 3.00 ± 0.17 s^-1^ (n = 36) | 4.7·10^-6^ (Paired t-test) |

**Parameter values Figure 6B**

|  | -PDBu | +PDBu | p-value |
| --- | --- | --- | --- |
| $E_{a}(0M)$ | 0.0 ± 0.2 RT | -0.7 ± 0.3 RT | As in figure B |
| $E_{a}(0.2M)$ | -4.6 ± 0.3 RT | -5.0 ± 0.3 RT | As in figure B |
| $E_{a}(0.3M)$ | -5.7 ± 0.2 RT | -6.0 ± 0.2 RT | As in figure B |
| $E_{a}(0.4M)$ | -7.0 ± 0.1 RT | -7.2 ± 0.1 RT | As in figure B |
| $E_{a}(0.5M)$ | -7.2 ± 0.1 RT | -7.5 ± 0.1 RT | As in figure B |

**Parameter values Figure 6C**

|  | Diff. in $k_{2,max}$ | Bootstrapped 95%CI |
| --- | --- | --- |
| $k_{2,max}(0M)$ | 1.70·10^-3^ s^-1^ | [1.1, 2.6]·10^-3^ s^-1^ |
| $k_{2,max}(0.2M)$ | 7.21·10^-2^ s^-1^ | [-0.17, 1.70]·10^-1^ s^-1^ |
| $k_{2,max}(0.3M)$ | 1.49·10^-1^ s^-1^ | [0.32, 2.71]·10^-1^ s^-1^ |
| $k_{2,max}(0.4M)$ | 3.83·10^-1^ s^-1^ | [0.88, 6.41]·10^-1^ s^-1^ |
| $k_{2,max}(0.5M)$ | 7.10·10^-1^ s^-1^ | [4.59, 9.66]·10^-1^ s^-1^ |

**Parameter values Figure 6D**

|  | Diff. in $E_{a}$ |
| --- | --- |
| $E_{a}(0M)$ | -0.69 RT |
| $E_{a}(0.2M)$ | -0.34 RT |
| $E_{a}(0.3M)$ | -0.25 RT |
| $E_{a}(0.4M)$ | -0.18 RT |
| $E_{a}(0.5M)$ | -0.27 RT |

**Parameter values Figure 6E**

| Parameter | Mean | Std | 95% CI | Unit | Mean (± SEM) (fig 7) |
| --- | --- | --- | --- | --- | --- |
| $k_{2,max}(0M,-PDBu)$ | 1.7·10^-3^ | 0.4·10^-3^ | [1.1, 2.5]·10^-3^ | 1/s | 1.70 ± 0.36·10^-3^ 1/s  (n=19) |
| $k_{2,max}(0.2M,-PDBu)$ | 1.78·10^-1^ | 0.48·10^-1^ | [0.98, 2.86]·10^-1^ | 1/s | 1.77 ± 0.50·10^-1^ 1/s  (n=20) |
| $k_{2,max}(0.3M,-PDBu)$ | 5.31·10^-1^ | 0.82·10^-1^ | [3.80, 7.04]·10^-1^ | 1/s | 5.31 ± 0.84·10^-1^ 1/s  (n=18) |
| $k_{2,max}(0.4M,-PDBu)$ | 1.90 | 0.18 | [1.56, 2.26] | 1/s | 1.91 ± 0.18 1/s  (n=22) |
| $k_{2,max}(0.5M,-PDBu)$ | 2.30 | 0.12 | [2.06, 2.54] | 1/s | 2.29 ± 0.13 1/s  (n=36) |
|  |  |  |  |  |  |
| $k_{2,max}(0M,+PDBu)$ | 3.4·10^-3^ | 0.7·10^-3^ | [2.3, 5.1]·10^-3^ | 1/s | 3.40 ± 0.76·10^-3^ 1/s  (n=19) |
| $k_{2,max}(0.2M,+PDBu)$ | 2.50·10^-1^ | 0.69·10^-1^ | [1.41, 3.99]·10^-1^ | 1/s | 2.49 ± 0.69·10^-1^ 1/s  (n=20) |
| $k_{2,max}(0.3M,+PDBu)$ | 6.82·10^-1^ | 1.07·10^-1^ | [4.86, 9.03]·10^-1^ | 1/s | 6.81 ± 1.11·10^-1^ 1/s  (n=18) |
| $k_{2}(0.4M,+PDBu)$ | 2.29 | 0.19 | [1.92, 2.68] | 1/s | 2.29 ± 0.20 1/s  (n=22) |
| $k_{2,max}(0.5M,+PDBu)$ | 3.01 | 0.17 | [2.68, 3.33] | 1/s | 3.00 ± 0.17 1/s  (n=36) |
|  |  |  |  |  |  |
| $k_{2,max}\left( 0M,+PDBu \right)-k_{2,max}(0M,-PDBu)$ | 1.7·10^-3^ | 0.4·10^-3^ | [1.1, 2.6]·10^-3^ | 1/s | 1.70·10^-3^ 1/s (n=19) |
| $k_{2,max}(0.2M,+PDBu)- k_{2,max}(0.2M,-PDBu)$ | 0.73·10^-1^ | 0.48·10^-1^ | [-0.17, 1.70]·10^-1^ | 1/s | 7.21·10^-2^ 1/s  (n=20) |
| $k_{2,max}(0.3M,+PDBu)- k_{2,max}(0.3M,-PDBu)$ | 1.49·10^-1^ | 0.61·10^-1^ | [0.32, 2.71]·10^-1^ | 1/s | 1.49·10^-1^ 1/s  (n=18) |
| $k_{2,max}(0.4M,+PDBu)- k_{2,max}(0.4M,-PDBu)$ | 3.82·10^-1^ | 0.14 | [0.88, 6.41]·10^-1^ | 1/s | 3.83·10^-1^ 1/s  (n=22) |
| $k_{2,max}(0.5M,+PDBu)- k_{2,max}(0.5M,-PDBu)$ | 7.09·10^-1^ | 0.13 | [4.59, 9.66]·10^-1^ | 1/s | 7.10·10^-1^ 1/s  (n=36) |

**Parameter values bootstrap analysis Figure 6**

|  | -PDBu | +PDBu | p-value |
| --- | --- | --- | --- |
| $k_{1}D$ | 140 ± 17 pA (n = 36) | 152 ± 20 pA (n = 36) | > 0.05 (Paired t-test) |

**Parameter values Figure 6-figure supplement 3A**

|  | -PDBu | +PDBu | p-value |
| --- | --- | --- | --- |
| $k_{-1}$ | 8.5 ± 0.6·10^-2^ s^-1^ (n = 36) | 8.5 ± 0.8·10^-2^ s^-1^ (n = 36) | > 0.05 (Paired t-test) |

**Parameter values Figure 6-figure supplement 3B**

|  | -PDBu | +PDBu | p-value |
| --- | --- | --- | --- |
| $RRP$ | 1.65 ± 0.14 nC (n = 36) | 1.69 ± 0.13 nC (n = 36) | > 0.05 (Paired t-test) |

**Parameter values Figure 6-figure supplement 3C**

| Model parameter | Value (unit) |
| --- | --- |
| $k_{1}$ | 0.14 (s^-1^) |
| $k_{-1}$ | 0.09 (s^-1^) |
| $D$ | 1.0 (nC) |
| $R$ | 1.6 (nC) |
|  |  |
| Sucrose function parameter |  |
| $k_{2,max}$ | 0.03-10 (s^-1^) |
| $t_{del}$ | 1.5 (s) |
| $\tau$ | 0.27 (s) |
| Duration of sucrose pulse | 7 (s) |

**Parameter values Figure 6-figure supplement 3D**

| Parameter | Mean | Std | 95% CI | Unit | Mean ± SEM (fig 7S3) |
| --- | --- | --- | --- | --- | --- |
| $k_{1}D(0.5M,-PDBu)$ | 140 | 16 | [110, 174] | pA | 140 ± 17 pA  (n=36) |
| $k_{1}D(0.5M,+PDBu)$ | 152 | 19 | [115, 192] | pA | 152 ± 20 pA  (n=36) |
|  |  |  |  |  |  |
| $k_{-1}(0.5M,-PDBu)$ | 8.5·10^-2^ | 0.6·10^-2^ | [7.4, 9.8]·10^-2^ | 1/s | 8.5 ± 0.6·10^-2^ 1/s  (n=36) |
| $k_{-1}(0.5M,+PDBu)$ | 8.5·10^-2^ | 0.8·10^-2^ | [7.0, 10.0]·10^-2^ | 1/s | 8.5 ± 0.8·10^-2^ 1/s  (n=36) |
|  |  |  |  |  |  |
| $RRP(0.5M,-PDBu)$ | 1647.3 | 141.2 | [1389.6, 1938.7] | pC | 1.65 ± 0.14 nC  (n=36) |
| $RRP(0.5M,+PDBu)$ | 1687.0 | 126.5 | [1446.5, 1938.6] | pC | 1.69 ± 0.13 nC  (n=36) |

**Parameter values bootstrap analysis Figure 6-figure supplement 3**
