# Supplementary material for: Additive effects on the energy barrier for synaptic vesicle fusion cause supralinear effects on the vesicle fusion rate
Source: eLife. 2015 Apr 14;4:e05531. doi: 10.7554/eLife.05531 (PMC4426983; doi:10.7554/eLife.05531)
Supplement: Figure 7—source data 1. — DOI: http://dx.doi.org/10.7554/eLife.05531.028 [file elife05531s007.docx]

**Figure 7-source data 1**

|  | Control | Cpx KO | p-value |
| --- | --- | --- | --- |
| $k_{2,max}(0M)$ | 7.8 ± 0.7·10^-4^ s^-1^ (n = 26) | 7.0 ± 1.6·10^-4^ s^-1^ (n = 47) | 1.0·10^-3^ (Mann-Whitney U test) |
| $k_{2,max}(0.25M)$ | 2.0 ± 0.2·10^-1^ s^-1^ (n = 24) | 8.4 ± 0.9·10^-2^ s^-1^ (n = 32) | 1.9·10^-8^ (Two-sample t-test) |
| $k_{2,max}(0.5M)$ | 3.3 ± 0.2 s^-1^ (n = 26) | 2.4 ± 0.1 s^-1^ (n = 47) | 2.6·10^-5^ (Two-sample t-test) |

**Parameter values Figure 7B**

|  | Control | Cpx KO | p-value |
| --- | --- | --- | --- |
| $E_{a}(0M)$ | 0.0 ± 0.1 RT | 0.1 ± 0.3 RT | As in figure B |
| $E_{a}(0.25M)$ | -5.6 ± 0.1 RT | -4.7 ± 0.1 RT | As in figure B |
| $E_{a}(0.5M)$ | -8.4 ± 0.1 RT | -8.0 ± 0.0(4) RT | As in figure B |

**Parameter values Figure 7C**

|  | Diff. in $k_{2,max}$ | Bootstrapped 95%CI |
| --- | --- | --- |
| $k_{2,max}(0M)$ | 7.23·10^-5^ s^-1^ | [-2.9, 3.8]·10^-4^ s^-1^ |
| $k_{2,max}(0.25M)$ | 1.16·10^-1^ s^-1^ | [0.81, 1.53]·10^-1^ s^-1^ |
| $k_{2,max}(0.5M)$ | 8.99·10^-1^ s^-1^ | [0.46, 1.36] s^-1^ |

**Parameter values Figure 7D**

|  | Diff. in $E_{a}$ |
| --- | --- |
| $E_{a}(0mM)$ | 0.10 RT |
| $E_{a}(0.25M)$ | 0.87 RT |
| $E_{a}(0.5M)$ | 0.32 RT |

**Parameter values Figure 7E**

| Parameter | Mean | Std | 95% CI | Unit | Mean ± SEM (fig 8) |
| --- | --- | --- | --- | --- | --- |
| $k_{2,max}(0M, Control)$ | 7.76·10^-4^ | 0.70·10^-4^ | [6.47, 9.19]·10^-4^ | 1/s | 7.8 ± 0.7·10^-4^ 1/s  (n=26) |
| $k_{2,max}(0.25M,Control)$ | 2.00·10^-1^ | 0.16·10^-1^ | [1.71, 2.32]·10^-1^ | 1/s | 2.0 ± 0.2·10^-1^ 1/s  (n=24) |
| $k_{2,max}(0.5M,Control)$ | 3.30 | 0.21 | [2.91, 3.74] | 1/s | 3.3 ± 0.2 1/s  (n=26) |
|  |  |  |  |  |  |
| $k_{2,max}(0M,Cpx KO)$ | 7.03·10^-4^ | 1.58·10^-4^ | [4.36, 10.0]·10^-4^ | 1/s | 7.0 ± 1.6·10^-4^ 1/s  (n=47) |
| $k_{2,max}(0.25M,Cpx KO)$ | 8.38·10^-2^ | 0.93·10^-2^ | [6.62, 10.3]·10^-2^ | 1/s | 8.4 ± 0.9·10^-2^ 1/s  (n=32) |
| $k_{2,max}(0.5M,Cpx KO)$ | 2.40 | 0.09 | [2.24, 2.58] | 1/s | 2.4 ± 0.1 1/s  (n=47) |
|  |  |  |  |  |  |
| $k_{2,max}\left( 0M,Cpx KO \right)-k_{2,max}(0M,Control)$ | 7.32·10^-5^ | 1.72·10^-4^ | [-2.9, 3.8]·10^-4^ | 1/s | 7.23·10^-5^ 1/s |
| $k_{2,max}\left( 0.25M,Cpx KO \right)-k_{2,max}(0.25M,Control)$ | 1.16·10^-1^ | 0.18·10^-1^ | [0.81, 1.53]·10^-1^ | 1/s | 1.16·10^-1^ 1/s |
| $k_{2,max}\left( 0.5M,Cpx KO \right)-k_{2,max}(0.5M,Control)$ | 8.95·10^-1^ | 2.30·10^-1^ | [0.46, 1.36] | 1/s | 8.99·10^-1^ 1/s |

**Parameter values Bootstrap analysis Figure 7**

|  | Control | Cpx KO | p-value |
| --- | --- | --- | --- |
| $k_{1}D$ | 133 ± 25 pA (n = 26) | 156 ± 21 pA (n = 47) | > 0.05 (Two-sample t-test) |

**Parameter values Figure 7-figure supplement 1A**

|  | Control | Cpx KO | p-value |
| --- | --- | --- | --- |
| $k_{-1}$ | 8.3 ± 1.2·10^-2^ s^-1^ (n = 26) | 8.9 ± 0.8·10^-2^ s^-1^ (n = 47) | > 0.05 (Two-sample t-test) |

**Parameter values Figure 7-figure supplement 1B**

|  | Control | Cpx KO | p-value |
| --- | --- | --- | --- |
| $RRP$ | 1.57 ± 0.15 nC (n = 26) | 1.65 ± 0.15 nC (n = 47) | > 0.05 (Two-sample t-test) |

**Parameter values Figure 7-figure supplement 1C**

| Model parameter | Value (unit) |
| --- | --- |
| $k_{1}$ | 0.75 (s^-1^) |
| $k_{-1}$ | 0.09 (s^-1^) |
| $D$ | 1.4 (nC) |
| $R$ | 1.6 (nC) |
|  |  |
| Sucrose function parameter |  |
| $k_{2,max}$ | 0.01-10 (s^-1^) |
| $t_{del}$ | 1.2 (s) |
| $\tau$ | 0.14 (s) |
| Duration of sucrose pulse | 4 (s) |

**Parameter values Figure 7-figure supplement 1D**

| Parameter | Mean | Std | 95% CI | Unit | Mean ± SEM (fig S6) |
| --- | --- | --- | --- | --- | --- |
| $k_{1}D(0.5M, Control)$ | 134 | 24 | [91, 184] | pA | 133 ± 25 pA  (n=26) |
| $k_{1}D(0.5M,Cpx KO)$ | 155 | 21 | [115, 198] | pA | 156 ± 21 pA  (n=47) |
|  |  |  |  |  |  |
| $k_{-1}(0.5M, Control)$ | 8.4·10^-2^ | 1.1·10^-2^ | [6.3, 10.7]·10^-2^ | 1/s | 8.3 ± 1.2·10^-2^ 1/s  (n=26) |
| $k_{-1}(0.5M,Cpx KO)$ | 8.9·10^-2^ | 0.8·10^-2^ | [7.3, 10.4]·10^-2^ | 1/s | 8.9 ± 0.8·10^-2^ 1/s  (n=47) |
|  |  |  |  |  |  |
| $RRP(0.5M, Control)$ | 1558.1 | 150.1 | [1268.3, 1855.1] | pC | 1.57 ± 0.15 nC  (n=26) |
| $RRP(0.5M,Cpx KO)$ | 1646.9 | 149.4 | [1363.4, 1950.5] | pC | 1.65 ± 0.15 nC  (n=47) |

**Parameter values Bootstrap analysis Figure 7-figure supplement 1**
