# Supplementary material for: Additive effects on the energy barrier for synaptic vesicle fusion cause supralinear effects on the vesicle fusion rate
Source: eLife. 2015 Apr 14;4:e05531. doi: 10.7554/eLife.05531 (PMC4426983; doi:10.7554/eLife.05531)
Supplement: Source code 1. — Custom software to analyze HS-induced postsynaptic currents written in MATLAB (only compatible with MATLAB R2013 or older). Instructions for how to use the program are in the readme file. Use on a Mac or Linux system requires specification of the location of the poi_library when asked for by the program. DOI: http://dx.doi.org/10.7554/eLife.05531.031 [file elife05531s008.zip › doc/conversion.html]

index


# File conversion

## Goal of conversion functions

The current version of Sucrose Analysis is only capable of loading files generated by Axon instruments with an *.ABF* extension. Currently it also supports only files that contain a single concentration per file. In order to overcome these limitations, a conversion option has been built in to allow the user to write his or her own conversion function. The conversion function will allow the user free access to convert any valid file into a session file that can be used for analysis.

## Prerequisites

A conversion file requires specific in and outputs to be passed to the conversion function. When writing the conversion function, make sure you check out the structure of the session file first to make sure all the relevant fields are generated in your conversion function. The program will verify whether all required fields are present after the session file is generated, but it will not test its content.

## Outline of a conversion function

A conversion function should have two inputs: a file container version and a cell array containing the filenames to be processed. The former is used to verify the data structure and make sure it is compatible with the version of the software the user is running. In the conversion function, it can be used to retrieve an empty data structure with the basic fields inserted using the *createDataStructDefinition()* function. This function takes the file container version as input and returns the empty data structure with required fields. The cell array of files should contain the data files that need to be processed. It is strongly advised to use the full path of the files to prevent any issues with the current path.

The conversion should return two outputs: a boolean to indicate whether conversion was successfull and the generated data structure. The boolean is only needed to indicate success and is used to display to the user that conversion was successfull. The data structure that is returned will be stored in a session file and will become available for analysis in the main interface. After the conversion function has finished, the structure will be verified to make sure it has the correct structure. If verification fails, a message is inserted in the log and the success boolean is changed to false.

The conversion implementation itself is entirely up to the user, but note that some points need to be considered. Firstly, the filename field currently requires that it ends with the suffix *[0-9]\{4}.abf*, meaning that the filename needs to end with 4 digits and the *.abf* extension. This is necessary for proper linking to occur in the main interface, with the suffix being unique among all filenames, and the prefix being unique to all files that need to be linked (see also the Usage page). The conversion should also create two rawdata fields: one with the real raw data (*rawdata*), and one with an optional correction applied (*rawdataCorr*). The corrected data will be used for data fitting and must have a starting baseline around zero for fitting to be successfull. Both the groupname and concentration fields must be set properly for each entry in the data structure: these are used to separate the maximal and submaximal responses and to determine the linking and parameter order. The groupname should be identical to the concentration, but with "mM" appended. The concentration should be a scalar value, while the groupname should be a *char*.

Finally, a structure containing the filters should be created by the conversion function. Currently, the only filter required is the block filter (*blck\_fltrs*). This filter contains the location for each block that is present in the data entry: it should be a matrix of n × b, where n is the number of samples and b is the number of blocks. The filter for each block should be true during the entire sucrose pulse of that block, and false everywhere else. In the main interface, loading of files is limited to two blocks only, but in the conversion function there is no limit to the number of blocks within reason. The analysis routines are also not limited to two blocks, so if more than two blocks need to be analyzed a custom conversion function is required. The number of columns in the block filter should correspond to the value of *nrOfBlocks* in the data structure.

Back to top

## Example conversion function

The following matlab code shows an example conversion function that returns a valid data structure. This example also shows some examples of input checks and basic parameter initialization (see lines 26 and 27): when using return to prematurely exit the conversion function, make sure the return values are set to empty and false. In order to append information to the log, use the function *appendSucroseLog()*. This function requires at least one parameter containing a string with the message, the second parameter is a keyword that indicates the severity of the event. The following values are implemented:

- 'info': green color
- 'abnormal': blue/gray color
- 'urgent': orange color
- 'critical': red color

Any other keyword value will be ignored and the entry will be indicated in black. It is also possible to supply an exception structure directly as the log message, which will automatically label the event as 'urgent' in the log. Whenever handling files within a file system, the use of the **try … catch** structure is strongly advised to help with recovery after errors (see lines 97 … 113 for an example).

```
1   function [bSuccess, dataStruct] = extractSucrosePulses(fileContainerVer, dataFiles)
2   %EXTRACTSUCROSEPULSES Example of a user-defined function for creating a
3   %session file suitable for loading in Sucrose Analysis
4   %
5   %   This file shows how to create a function for converting
6   %   electrophysiology data to a format that can be used as input for
7   %   sucrose analysis. This template can be extended or replicated for use
8   %   within Sucrose Analysis. It can be called from within the main
9   %   interface via the file menu. The applyUserConversion allows the
10  %   selection of this file. It also handles the saving of the data
11  %   structure and the verification of the structure.
12  %
13  %   input (required): 
14  %       fileContainerVer:   the version of the output file
15  %       dataFiles:          cell array of file names for importing
16  %   output (required):
17  %       bSuccess:   indicates whether conversion was succesfull
18  %       dataStruct: the finished structure to be used by Sucrose Analysis
19  %
20  %See also: applyUserConversion.m, createDataStructDefinition.m,
21  %verifyDataStructure.m
22  
23  %#ok<*AGROW>
24  
25  %default values for return parameters
26  bSuccess = false;
27  dataStruct = [];
28  
29  %basic checks
30  if nargin ~= 2
31      return;
32  end
33  
34  %{
35      First, create an empty data structure based on the container version
36      supplied to this function. As the software develops, the data structure
37      will change. This will cause the list of defintions provided by
38      createDataStructDefintion to grow as well. The definition will always be
39      equal to the version used by the analysis program.
40  %}
41  dataStruct = createDataStructDefinition(fileContainerVer);
42  if isempty(dataStruct)
43      %unsupported data container format
44      appendSucroseLog('Unsupported data container format', 'urgent');
45      return;
46  end
47  
48  %check the format of dataFiles
49  if isempty(dataFiles) || ~iscell(dataFiles) 
50      appendSucroseLog('No valid files supplied', 'urgent');
51      return;
52  end
53  
54  %{
55      for each file use the appropriate load function to collect the data. Note
56      that in this example only abf files are processed using the abfload
57      function from the file exchange. If you wish to support more than 1 file
58      type, expand the case list with the correct file extension (including
59      the period!) for each supported file type. Otherwise, create a specifc
60      conversion function for each file type.
61  %}
62  for f = 1:numel(dataFiles)
63      %test if the data file exists
64      file = dataFiles{f};
65      if exist(file, 'file') ~= 2
66          %it does not exist so skip
67          continue;
68      end
69      
70      %{
71          Set the insertion index according to the current size. When an
72          empty data set is generated, the number of elements in dataStruct
73          is 1, but the filename field will be empty. If this is the case,
74          then the insertion index should be 1, otherwise it should be the
75          last index + 1.
76      %}
77      if isempty(dataStruct(1).filename)
78          insertIdx = 1;
79      else
80          insertIdx = numel(dataStruct) + 1;
81      end
82      
83      %split the file name into its components
84      [path, file, ext] = fileparts(file);
85      
86      switch(ext)
87          case '.abf'
88              %{
89                  load the file using abfload and retrieve the data, sample
90                  interval and the header. In the case of abfload, all
91                  required information about gain and recording units can
92                  be extracted from the header information. Make sure
93                  there is only one column for the data in the final
94                  structure, as multiple sweeps are not supported by the
95                  analysis program at this time.
96              %}
97              try
98                  [data, sample_int, hdr] = abfload(fullfile(path, [file ext]));
99                  if sample_int > 0
100                     %convert to seconds
101                     sample_int = sample_int/1e6;
102                 end
103                 if size(data, 2) > 1
104                     data = data(:,1);
105                 end
106             catch me
107                 disp(getReport(me, 'basic'));
108                 errordlg({'The function abfload was not on the matlab search path.'; 
109                           'Please download it from the matlab fileexchange or add';
110                           'it to the search path.'}, 'Data import failed');
111                 appendSucroseLog('Function abfload was missing', 'urgent');
112                 return;
113             end
114             
115             try
116                 %{
117                     retrieve the scaling info from the header: the program
118                     expects the data to be expressed in pA so for some files a
119                     scaling factor is required. Such a list can be expanded
120                     if necessary to support more scalings. The program
121                     expects that data is recorded in voltage-clamp mode,
122                     meaning the data is measured in amperes
123                 %}
124                 scalingVals = unique(hdr.recChUnits);
125                 idx = regexp(scalingVals, '.A', 'once');
126                 idx = ~cellfun('isempty', idx);
127                 idx = find(idx, 1, 'first');
128                 scalingVal = scalingVals{idx};
129                 
130                 switch(scalingVal)
131                     case 'pA'
132                         scaleFactor = 1;
133                     case 'nA'
134                         scaleFactor = 1e3;
135                     otherwise
136                         %no valid scaling found, skip file
137                         continue;
138                 end
139                 
140                 %{
141                     retrieve the gain value from the header: the data should
142                     be set at gain level 1, so any other value requires
143                     correction
144                 %}
145                 signalGain = hdr.fSignalGain;
146                 signalGain = signalGain(1);
147             catch me2
148                 disp(getReport(me2, 'basic'));
149                 errordlg({'The function abfload returned an incomplete or bad header.';
150                           'Please make sure your abf file is not damaged.'},...
151                           'Data import failed');
152                 appendSucroseLog('Header information was incorrect', 'urgent');
153                 return;
154             end
155             
156             %{
157                 process the data according to the protocol used for
158                 acquisition: in this case the data is split into two equal
159                 parts and the block is defined between a quarter and half the
160                 duration of each data part. Values for these parameters can be
161                 hard-coded or defined via input dialogs. This is just an
163                 example of how to load and convert the data. For fitting,
164                 please ensure that the values for the blocks (start time
165                 and duration) are set correctly.
166             %}
167             data250 = (data(1:end/2)*scaleFactor)/signalGain;
168             data500 = (data(end/2+1:end)*scaleFactor)/signalGain;
169             
170             %create the filter data
171             filterData250 = false(numel(data250),1);
172             filterData250(floor(numel(data250)*0.25):floor(numel(data250)*0.50)) = true;
173             filterData500 = false(numel(data500),1);
174             filterData500(floor(numel(data500)*0.25):floor(numel(data500)*0.50)) = true;
175             
176             %{
177                 update the filenames to include the necessary marker at the
178                 end for sucrose analysis to enable linking. Linking of
179                 files is done by comparing the filename before the final 4
180                 digits: identical names will be assumed to belong to a
181                 linked file. The method used here assures that when using a
182                 single file for both conditions, a unique name is generated
183                 which can be used for linking.
184             %}
185             file     = [file 'a_0001']; 
186             linkfile = [file 'a_0002'];
187             
188             %{
189                 Fill in the structure for submax. In this example, the
190                 rawdataCorr field uses the same data as the rawdata field.
191                 The purpose of the rawdataCorr field is to store data
192                 corrected for baseline leak and baseline drift. Ideally the
193                 data for fitting should start at 0.
194             %}
195             dataStruct(insertIdx).filename           = [file ext];
196             dataStruct(insertIdx).linkedFile         = {[linkfile ext]};
197             dataStruct(insertIdx).pathname           = path;
198             dataStruct(insertIdx).groupname          = '250mM';
199             dataStruct(insertIdx).genotype           = 'WT';
200             dataStruct(insertIdx).concentration      = 250;
201             dataStruct(insertIdx).rawdata            = data250;
202             dataStruct(insertIdx).rawdataCorr        = data250;
203             dataStruct(insertIdx).nrOfBlocks         = 1;
204             dataStruct(insertIdx).sample_int         = sample_int;
205             dataStruct(insertIdx).gain               = signalGain;
206             dataStruct(insertIdx).filters.blck_fltrs = filterData250;
207             
208             %fill in the structure for 500
209             dataStruct(insertIdx+1).filename           = [linkfile ext];
210             dataStruct(insertIdx+1).linkedFile         = {[file ext]};
211             dataStruct(insertIdx+1).pathname           = path;
212             dataStruct(insertIdx+1).groupname          = '500mM';
213             dataStruct(insertIdx+1).genotype           = 'WT';
214             dataStruct(insertIdx+1).concentration      = 500;
215             dataStruct(insertIdx+1).rawdata            = data500;
216             dataStruct(insertIdx+1).rawdataCorr        = data500;
217             dataStruct(insertIdx+1).nrOfBlocks         = 1;
218             dataStruct(insertIdx+1).sample_int         = sample_int;
219             dataStruct(insertIdx+1).gain               = signalGain;
220             dataStruct(insertIdx+1).filters.blck_fltrs = filterData500;
221         otherwise
222             %skip this file
223             continue;
224     end
225     
226 end
227 
228 %finished with the data structure
229 if ~isempty(dataStruct)
230     bSuccess = true;
231 end
232 
233 end
```

Please note that the returned structure may not be suitable for fitting, as the splitting of the file happens at arbitrary points, and the corrected data is identical to the raw data (which may not start at zero). In this example, a switch statement (line 86) is used to allow the possibility of mixed file types to be loaded, which is allowed but not recommended. Also note that when the user specifies the conversion function, it is temporarily added to the Matlab search path to enable its use. This means that any functions called from within the conversion function should be located on the search path as well to make sure they can be called. It is the user's responsibility to add any required functions to the search path before running the conversion. A good practice is to add conversion functions to the conversion folder within the application folder, as this folder is added to the search path at runtime if it is not already present. If the conversion function starts with **extract**, it will be preloaded in a list dialog of conversion functions. This conversion list will also remember the previous conversion function that was used, allowing for quicker conversion.

Back to top
