# Supplementary material for: Additive effects on the energy barrier for synaptic vesicle fusion cause supralinear effects on the vesicle fusion rate
Source: eLife. 2015 Apr 14;4:e05531. doi: 10.7554/eLife.05531 (PMC4426983; doi:10.7554/eLife.05531)
Supplement: Source code 1. — Custom software to analyze HS-induced postsynaptic currents written in MATLAB (only compatible with MATLAB R2013 or older). Instructions for how to use the program are in the readme file. Use on a Mac or Linux system requires specification of the location of the poi_library when asked for by the program. DOI: http://dx.doi.org/10.7554/eLife.05531.031 [file elife05531s008.zip › doc/export.html]

Exporting data


# Exporting data

After the data has been reviewed and bad cells have been removed from the data set, the results of the analysis can be exported to an Excel file. This step will function on all platforms, and will result in an Excel file with a separate worksheet for each genotype. Each worksheet will contain the fitted parameters, per sucrose pulse (´block´), of all analysed files for this genotype, sorted by sucrose concentration. The naming convention for these worksheets is "par\_[genotype]" (e.g. "par\_WT"). Furthermore, an 'Info' sheet is generated, which contains information on the raw data. Further analysis, such as calculation of mean and SEM values, as well as testing for statistical significance can be done in Excel.

## Fitting results

Typically, the structure of one worksheet in such an exported file is as follows:

|  |  | Block |  |  |  |  |  |  |  |  |
| --- | --- | --- | --- | --- | --- | --- | --- | --- | --- | --- |
| File name | Sucrose concentration (mM) | Priming rate k1 (1/s) | Unpriming rate k-1 (1/s) | Release rate constant k2max (1/s) | Onset delay tdel (s) | Rise time tau (s) | Depot pool D (pC) | Primed pool R (pC) | Cumulative release (pC) | Cumulative refill (pC) |

The leftmost column contains the file names, while the next column gives the sucrose concentration used in each of these recordings. All other columns contain the fitted parameters for a single sucrose pulse. Submaximal concentrations furthermore contain columns describing the cumulative release and the cumulative refill obtained from the fits. These values can be used to calculate the fraction of RRP depleted by the submaximal stimulus. For each subsequent sucrose pulse ('block'), all these columns - except for the ones containing the file names and sucrose concentrations - are repeated (with different fitted values, obviously) and headed by a 'Block' cell.

## The 'Info' sheet

This data sheet contains a summary of the raw data properties per data file, as well as the fit quality. Its structure is typically as follows:

|  |  |  | Block |  |  |  |  |  |  |  |
| --- | --- | --- | --- | --- | --- | --- | --- | --- | --- | --- |
| File name | Sucrose concentration (mM) | Genotype | Fitting method | Fit start (s) | Sucrose pulse (s) | Baseline current (pA) | Baseline slope (pA/s) | Noise level (pA) | Peak current (pA) | Fit quality (pA) |

For each subsequent sucrose pulse ('block'), all these columns - except for the ones containing the file names, sucrose concentrations and genotypes - are repeated and headed by a 'Block' cell.

- File name: the name of the (.abf) file containing the raw data.
- Sucrose concentration (mM): the sucrose concentration in mM.
- Genotype: the genotype.
- Fitting method: the fitting method used (local search/genetic/annealing).
- Fit start (s): the time point of fit start as set by the user (which usually coincides with the time when sucrose application started).
- Sucrose pulse (s): the duration of the sucrose pulse as set by the user.
- Baseline current (pA): average leak current during the [baseline length] seconds (default is 0.1s, see preferences section in Using the GUI) just preceding the above value of fit start.
- Baseline slope (pA/s): slope of the linear interpolation between the current at the start of the sucrose pulse and the current 2 seconds after the end of the pulse. The time point at the start of the pulse is equal to the above value of fit start, while the time point after the pulse is equal to (fit start + sucrose pulse + 2 seconds). This adaptive baseline can be used to correct the data (see preferences section in Using the GUI). If not used, the slope will be zero.
- Noise level (pA): the average distance between the raw data and the smoothed raw data. Smoothing is done by taking the moving average of 2000 samples. The noise level is calculated in the same way as the fit quality (see below), but with smoothi replacing fiti. The noise level can be roughly interpreted as the lowest attainable value of the fit quality (i.e. the quality of the fit is constrained by the noise level).
- Peak current (pA): the peak current of the smoothed raw data. In general, this value is less negative than the peak current of the raw data.
- Fit quality (pA): the average distance between the raw data and the fit. The fit quality is calculated as follows:where ndata is the number of data points used for fitting, datai the ith entry of the raw data, and fiti the ith entry of the fit.
