# Supplementary material for: Additive effects on the energy barrier for synaptic vesicle fusion cause supralinear effects on the vesicle fusion rate
Source: eLife. 2015 Apr 14;4:e05531. doi: 10.7554/eLife.05531 (PMC4426983; doi:10.7554/eLife.05531)
Supplement: Source code 1. — Custom software to analyze HS-induced postsynaptic currents written in MATLAB (only compatible with MATLAB R2013 or older). Instructions for how to use the program are in the readme file. Use on a Mac or Linux system requires specification of the location of the poi_library when asked for by the program. DOI: http://dx.doi.org/10.7554/eLife.05531.031 [file elife05531s008.zip › doc/intro.html]

Introduction


# Introduction

This program is used for fitting electrophysiological responses of neurons to superfusion with hypertonic sucrose. The biophysical model to which the data is fitted is briefly explained on the Theory page. Upon completion of the fitting procedure, all parameters, including RRP size and release rate constant, can be exported to an Excel file for further analysis.

The GUI can only load electrophysiological data recorded in Axon binary files (.abf). However, the user is free to write a script which processes a different type of data file and produces a data structure according to the design outlined on the Theory page. This data structure can subsequently be loaded into the GUI and used for fitting the data. Versions up to and including 0.7 build 0714.08 only work on Matlab release 2013b and earlier.
