# Supplementary material for: Additive effects on the energy barrier for synaptic vesicle fusion cause supralinear effects on the vesicle fusion rate
Source: eLife. 2015 Apr 14;4:e05531. doi: 10.7554/eLife.05531 (PMC4426983; doi:10.7554/eLife.05531)
Supplement: Source code 1. — Custom software to analyze HS-induced postsynaptic currents written in MATLAB (only compatible with MATLAB R2013 or older). Instructions for how to use the program are in the readme file. Use on a Mac or Linux system requires specification of the location of the poi_library when asked for by the program. DOI: http://dx.doi.org/10.7554/eLife.05531.031 [file elife05531s008.zip › doc/license.html]

License information


# License information

## Sucrose analysis

The main software (Sucrose Analysis) is covered under the Creative Commons BY-SA-NC license, version 4.0 with the following limitations:

- BY: Attribution
- SA: Share-Alike
- NC: Non-Commercial

For details and the full license text, look at the Creative Commons website. The biophysical model was developed by Niels Cornelisse, and the implementation of the model in Matlab was done by Bas Schotten. Interface design and data handling was implemented by Jurjen Broeke. Details about the software can be found on the CNCR website (at some point). Application of this model and the main software was published in [Schotten et al., 2014].

For attribution, the following information should be used:

```
                Center for Neurogenomics and Cognitive Research
                Neuroscience Campus Amsterdam
                Vrije Universiteit (VU) and VU medical center
                De Boelelaan 1085
                1081HV Amsterdam
                The Netherlands
```

## Third party code

Most of the third party code used in this project was retrieved from the Matlab FileExchange and is covered by the BSD license:

```
            Redistribution and use in source and binary forms, with or without
            modification, are permitted provided that the following conditions are
            met:
            
                * Redistributions of source code must retain the above copyright
                  notice, this list of conditions and the following disclaimer.
                * Redistributions in binary form must reproduce the above copyright
                  notice, this list of conditions and the following disclaimer in
                  the documentation and/or other materials provided with the distribution
            
            THIS SOFTWARE IS PROVIDED BY THE COPYRIGHT HOLDERS AND CONTRIBUTORS "AS IS"
            AND ANY EXPRESS OR IMPLIED WARRANTIES, INCLUDING, BUT NOT LIMITED TO, THE
            IMPLIED WARRANTIES OF MERCHANTABILITY AND FITNESS FOR A PARTICULAR PURPOSE
            ARE DISCLAIMED. IN NO EVENT SHALL THE COPYRIGHT OWNER OR CONTRIBUTORS BE
            LIABLE FOR ANY DIRECT, INDIRECT, INCIDENTAL, SPECIAL, EXEMPLARY, OR
            CONSEQUENTIAL DAMAGES (INCLUDING, BUT NOT LIMITED TO, PROCUREMENT OF
            SUBSTITUTE GOODS OR SERVICES; LOSS OF USE, DATA, OR PROFITS; OR BUSINESS
            INTERRUPTION) HOWEVER CAUSED AND ON ANY THEORY OF LIABILITY, WHETHER IN
            CONTRACT, STRICT LIABILITY, OR TORT (INCLUDING NEGLIGENCE OR OTHERWISE)
            ARISING IN ANY WAY OUT OF THE USE OF THIS SOFTWARE, EVEN IF ADVISED OF THE
            POSSIBILITY OF SUCH DAMAGE.
```

with specific copyright statements added below for each function. The license text is also included in the **license.txt** file in the software package.

### enableDisableFig

Developed by Yair Altman and published on the Matlab FileExchange as file ID 15895 with the following copyright information:

Copyright © 2011, Yair Altman   
All rights reserved.

  
The code of this function was not modified for this project.

### abf2load

Developed by Forrest Collman and Harald Hentschke and published on the Matlab FileExchange as file ID 22114, with the following copyright information:

Copyright © 2009, Forrest Collman   
Copyright © 2004, Harald Hentschke   
All rights reserved.

  
The code of this function was not modified for this project.

### xlwrite

Developed by Alec de Zegher and published on the Matlab FileExchange as file ID 38591, with the following copyright information:

Copyright © 2013, Alec de Zegher   
All rights reserved.

  
The code of this function was not modified for this project. This function requires the Apache POI library, which can be downloaded from their website. This software was successfully tested with version *poi-3.8-20120326* on 64bit Linux systems and Mac OSX platforms.

### progressbar

Developed by Steve Hoelzer and published on the Matlab FileExchange as file ID 6922, with the following copyright information:

Copyright © 2005, Steve Hoelzer   
All rights reserved.

  
The code of this function was not modified for this project, but the name was changed to prevent conflicts with other programs.

### makeBarPlotSucrose

This function is a modified version of the makeBarPlot function developed by Arthur de Jong at the Vrije Universiteit Amsterdam. This function was not published before and falls under the same license as the main software.
