# Supplementary material for: Additive effects on the energy barrier for synaptic vesicle fusion cause supralinear effects on the vesicle fusion rate
Source: eLife. 2015 Apr 14;4:e05531. doi: 10.7554/eLife.05531 (PMC4426983; doi:10.7554/eLife.05531)
Supplement: Source code 1. — Custom software to analyze HS-induced postsynaptic currents written in MATLAB (only compatible with MATLAB R2013 or older). Instructions for how to use the program are in the readme file. Use on a Mac or Linux system requires specification of the location of the poi_library when asked for by the program. DOI: http://dx.doi.org/10.7554/eLife.05531.031 [file elife05531s008.zip › doc/theory.html]

Theory


# Theory

## Biophysical model used for fitting

The main purpose of this program is to fit hypertonic sucrose (HS)-induced EPSCs to a vesicle state model, as explained in Schotten et al., unpublished [ref].

Briefly, this model assumes application of hypertonic sucrose to selectively lower the amount of energy required for vesicle fusion, thus depleting a pool of primed vesicles with a concentration-dependent release rate (k2, see figure below).

By fitting the HS-induced EPSCs, one obtains the maximal value of this release rate constant (k2) during HS application, the size of the primed pool (R), the priming rate constant (k1), the unpriming rate constant (k-1) and the size of the upstream non-primed depot pool (D). Using a result from transition state theory, the Eyring equation, the fitted release rates can be transformed into a fusion energy barrier height ΔE:

Where k2max is the maximal value of the fitted release rate constant in (1/s), the transmission coefficient κ is usually taken equal to 1, kB is Boltzmann's constant, T the absolute temperature in degrees K, h Planck's constant, and ΔE the fusion energy barrier height in J. (So, at T = 293K or 20°C, the factor preceding the exponential has value ~6.11e12.) Thus one can use the fits to infer the effect of genetic perturbations or biochemical manipulations on this energy barrier.

The change in release rate constant k2 upon sucrose application is clearly not instantaneous, as can be seen from sucrose-induced EPSCs. We model k2 as a function of time via an expo-exponential:

Here k2max is the maximal value of the release rate constant during sucrose application, t0 is the time when sucrose application starts, tdel is a parameter describing the delay in sucrose onset and τrise captures the speed of increase in release rate during sucrose application.

Back to top

## Session file and data structure

As explained in the Using the GUI section, one can save the
current session to a Matlab .mat-file. This session file contains the number 'versionNr' of the current
version of the software, a data structure 'dataStruct' used for storing the raw data and the fitting
results, and the version number 'fileVersion' of the current version of the data structure. If the value
of 'fileVersion' of a loaded session file does not match the value of 'fileVersion' in the program, the
session file cannot be loaded.

The format of the data structure is as shown in the middle and right columns in the following figure:

The number *n* of data structures *dataStruct* equals the number of raw data files loaded by the user. The contents of each *dataStruct* is as follows:

- blockAnalysis: each raw data file can currently contain up to 2 sucrose pulses, or 'blocks'. Each of these blocks is fitted in an independent manner, thus resulting in two separate structures.

- label: a string describing the fitting method used for the initial fit (genetic/annealing/local).
- label\_extended: a string describing the fitting method used for fitting the extended model (genetic/annealing/local).
- parameters: a vector containing the fitted model parameters. This vector is of the form [k1, k-1, [k2MaxConc], [k2SubmaxConc], D, R], where [k2MaxConc] = [k2max, tdel, tauRise] related to an RRP-depleting stimulus, while [k2SubmaxConc] = [k2max, tdel, tauRise] is related to a submaximal sucrose stimulus. There are three values [k2SubmaxConc] for each submaximal stimulus. The total number of parameters is thus 4+3\*(*m*+1), where *m* is the number of submaximal sucrose stimuli.
- parameters\_extended: a vector of the same form as 'parameters', but these result from fitting the extended model.
- cost: the value of the SSE (Sum of Squared Errors between raw data and model) for the above parameter set. In case of a linked file, it is equal to the sum of the individual SSEs.
- cost\_extended: same as cost, but this value results from fitting the extended model.
- useWeightedFit: a Boolean denoting whether this fitting result was obtained with (true) or without (false) weighting.

- filename: a string containing the name+file extension of the raw data file.
- linkedFile: a cell array containing the name+file extension of the raw data file(s) linked to the current data file.
- pathname: a string containing the full path to the current data file.
- groupname: a string consisting of the sucrose concentration value in mM and "mM" (e.g. "250mM").
- genotype: a string describing the genotype.
- concentration: a number describing the sucrose concentration in mM.
- rawdata: a vector containing the raw data in pA.
- rawdataCorr: a vector containing the raw data in pA, corrected for the shift in baseline before and after the sucrose pulse.
- nrOfBlocks: a number equal to the number of sucrose pulses in the current data file.
- sample\_int: a number describing the interval in seconds with which the raw data was sampled (e.g. 10kHz sampling <-> sample\_int = 10-4 s).
- gain: a number describing the gain value at which the raw data was recorded.
- filters: logical arrays that can be applied to the raw data to filter out particular subsets.

- blck\_fltrs: a logical array that is applied to the raw data to filter out the sucrose pulses. The model is fitted to this filtered data.

Back to top

## Fitting procedure

Once all data has been loaded and everything is set to start the fitting procedure, the user can select the fitting method under Edit->Preferences. In general, one of the global fitting methods (genetic or annealing) is recommended, to minimise the risk of getting stuck in a local optimum, which would result in a bad fit. Currently, running a global fit is always followed up by a local search (using the Nelder-Mead or downhill simplex method). On the other hand, a user-chosen local fit is only followed up by a local search provided the initial local fit did not converge to the optimum within the given number of iterations.

As mentioned in the Using the GUI section, the 'Preferences' menu contains an option to 'Use weighted fit'. This fitting method makes use of a weighting factor for each of the nSub submaximal sucrose stimuli in the calculation of the cost (SSE):

Here, 'max' and 'sub' refer to maximal and submaximal sucrose stimuli respectively, so 'nsub' is the number of submaximal concentrations. The number of data points in each of the traces is the same, and is denoted 'ndata'. While the weighting factor for the maximal stimulus is equal to 1 for all data points, the weighting factors wisub are not constant throughout the trace. From the start of the trace until the timepoint (after the peak has occurred) when the evoked current is halfway between its peak value and its refill phase, the weight is taken constant. The weight of the remaining part of the trace decays exponentially until it has value 0.10 at j = ndata (i.e. the 'refill phase' of submaximal stimuli is considered less important). The constant phase of the submaximal stimuli weights is multiplied by the floor value of the ratio of the peak release rates (peakmax/peakisub), while the decaying phase is valued < 1:

The first time a data set is fitted, the non-primed depot pool (D) is assumed to be non-depletable. Thus, the refill phase of the sucrose pulse, equal to k1\*D for RRP-depleting stimuli, is assumed to be constant - one can only infer the refill rate of the RRP in pA, not the priming rate *constant* k1 in 1/s. Subsequently, once this fit has been finished, the user can choose to refit the data with the extended model in which is D is finite by choosing Fit->Fit extended model. This refitting procedure takes all parameters resulting from the initial fit constant, except for k1 and D. This approach was opted for because the number of degrees of freedom with k1 and D as free parameters from the outset, in addition to all other model parameters, seems to be too large to get reliable fitting results.

By default, the GUI shows the fitting result based on the non-depletable depot pool assumption. To see a plot of the fit using the extended model, tick the box 'Extended model' in the main interface.

Back to top
