# Supplementary material for: Additive effects on the energy barrier for synaptic vesicle fusion cause supralinear effects on the vesicle fusion rate
Source: eLife. 2015 Apr 14;4:e05531. doi: 10.7554/eLife.05531 (PMC4426983; doi:10.7554/eLife.05531)
Supplement: Source code 1. — Custom software to analyze HS-induced postsynaptic currents written in MATLAB (only compatible with MATLAB R2013 or older). Instructions for how to use the program are in the readme file. Use on a Mac or Linux system requires specification of the location of the poi_library when asked for by the program. DOI: http://dx.doi.org/10.7554/eLife.05531.031 [file elife05531s008.zip › doc/usage.html]

Using the GUI


# Using the GUI

After opening the program, the interface can look as follows:

## Loading data

Files should be loaded into the program one condition at a time (i.e. files of a single sucrose concentration and genotype).

First, enter the genotype. To enable the loading of files, enter the sucrose concentration of the to-be-loaded files and press enter. Multiple files can be loaded at once by selecting the relevant files using the Shift key to select ranges or the Control key to select individual files. Note that it is possible to load files of the same concentration in multiple actions; the list of these newly loaded files will be appended to the existing one. After pressing OK, the user is prompted with the following screen:

This window asks the user for a set of parameters that holds for all data files that were selected to be loaded. First, the length of the sucrose pulse(s) has to be entered. All pulses in a loaded data set are assumed to be of the same length. Currently, the maximum number of pulses in a single file is limited to 2. Next, the user has to enter the starting time for each pulse. If there is only a one pulse per file, the second value has to be blank. After pressing OK, all selected data will be loaded and a progressbar will show the progress of loading. Once loading has finished, the interface could look as follows (depending on the program version):

If the loaded group was non-existent, its label will now have been added to the 'Group' popup menu (1). All files contained in the selected group are shown as a list under 'Files' (2). Selecting one of these files will display the data in the plot window (3).

Back to top

## Linking files

Once all files have been loaded, click the checkbox ´Files linked´ (4) as in the above figure to associate submaximal sucrose stimulations (e.g. 0.25M) with RRP-depleting sucrose stimulations. For WT neurons, 0.5M sucrose is typically sufficient to deplete the RRP. However, for some genotypes the fusion energy barrier can be increased, requiring a stronger stimulus (e.g. 1.0M) to deplete the RRP. Therefore, every submaximal stimulus is linked in a pairwise manner to an RRP-depleting stimulus. If more than two concentrations were applied to a single cell, all these recordings are fitted simultaneously.

This process of linking recordings is necessary, as fitting ´non-linked´ submaximal sucrose stimulations will result in wrong estimates of the RRP size and the release rate. It is important to note that this association is achieved via a particular comparison of filenames, namely according to the following format:

[prefix]0001.abf

The matching is performed by stripping off the extension (.abf) and the recording number (here: 0001), and subsequently matching the remaining [prefix] of the RRP-depleting data set with the [prefix] of the 'submaximal' data set. For instance, in the above figure, the to-be-matched [prefix] would be '130821\_WT\_c09\_'.

The user should determine which concentration to designate as 'RRP-depleting'. All recordings with lower concentrations than the maximal one will be considered 'submaximal'.

Back to top

## Saving and loading sessions

For future reference, it is possible to save the current session via File->Save session. This will store the current data structure, containing among others the raw data and fit results (see the Theory section for a description of the data structure), in a Matlab .mat-file.

Previously saved session files can be loaded via File->Load session. Note that the software version with which the session file was saved is key here - if the data structure employed by the program has changed between time of loading and time of saving, the session file cannot be loaded.

In order to load and analyse recordings in a format different from .abf-files, the user should write a script which processes these data and returns a data structure as specified in the theory section.

Back to top

## Preferences

The preferences menu (Edit->Preferences) enables the user to modify parameters related to the model, the way the data is loaded, and the procedure used to fit data to the model. The preferences menu can look as follows:

The 'Model parameters' shown on the left control particular properties of the model and the way data is handled by the program:

- Sucrose decay: this value controls the decay time constant (in s) of the simulated sucrose responses. It cannot be fitted (default: 0.15s).
- Baseline length (sec): this parameter sets which part of the raw data is used for determining the baseline of the response (default: 0.1s).

The user fix certain model parameters during a fit by ticking the 'Use fixed parameters' box. Doing so will prompt the user, when starting the fit, with a window asking which parameter(s) to keep fixed. Subsequently, this/these parameter(s) can be given a particular value, or (by leaving the field empty) the parameter(s) for the second pulse in a trace can be kept at the same value(s) as fitted from the first pulse. To start a fit using these settings, use Fit->Fit all - fitting a single pulse via 'Refit data' will not work with fixed parameters.

Control over the 'Experimental features' is switched off by default:

- Basal release rate: this is the value of the release rate constant k2 (in 1/s) before the start of sucrose application. It cannot be fitted (default: 0 1/s).
- Default gain: raw data could have been recorded at a gain value different from 1. When loading .abf-files, the gain value used during the recordings can be extracted. If not (e.g. when the user is using a different file type), the recorded current is multiplied by the factor entered here (default: 1). *Note that this option only applies when loading new data!*

The 'Fitting parameters' shown on the right control the fitting procedure:

- Fitting method: this popup menu contains the options 'local search', 'genetic' and 'annealing'. 'Local search' causes the fitting procedure to use a local search method (Nelder-Mead algorithm). The other two options activate a global search method, based on a genetic algorithm ('genetic') or on simulated annealing ('annealing').
- Fitting output: this popup menu contains the options 'final', 'off', 'iter' and 'diagnose'. 'Final' will cause Matlab to only show the end result of a fit in the command window; 'off' will turn off all output; 'iter' will show the result from each iteration during the fitting procedure; and 'diagnose' does the same as 'iter', but it also lists some problem information and the options that have been changed from the defaults.
- Evaluations/parameter: this parameter sets the maximum number of function evaluations per model parameter (default: 400).
- Iterations/parameter: this parameter sets the maximum number of iterations per model parameter (default: 400).
- Use random parameters: tick this checkbox to generate a set of random parameters for each fitted sucrose pulse.
- Use weighted fit: tick this checkbox to enable the use of a weighted fit. This will have the effect of submaximal stimuli being weighted more heavily in the calculation of the cost function, to make up for the fact that the currents in submaximal responses are smaller by default. The weighting is done in proportion to the ratio of peak release rates between the maximal and submaximal stimuli.
- Adaptive baseline: tick this checkbox to enable the use of a linearly interpolated baseline during a sucrose pulse. When this is switched off, the sucrose pulse is only shifted vertically by the current calculated using the (default, see above) 0.1s baseline before a pulse. Switching on this option will also take into account the baseline after the pulse and use this to linearly interpolate between both baseline values, causing the pulse to shift and 'rotate' upon baseline correction.

Pushing the 'Default' button will return the model or fitting parameters to their original values.

Back to top

## Fitting data

One can choose to either fit all data in one go (via Fit->Fit all), or (re)fit traces one by one by right-clicking on a trace in the 'Files' list and selecting 'Refit data'. See also the section about the fitting procedure on the Theory page.

Back to top

## Using the log

The software keeps track of the actions performed by the user via a log, which can be accessed via the Help menu (Help->Show log). The log keeps track of a number of actions/events, including the loading of raw data files; performance during the fitting procedure (one can for instance monitor the duration of a fit); manually changed parameters when reviewing fit results; possible errors/problems encountered during the fitting procedure; etc. The severity of a logged action is colour-coded, ranging from green to red. The log can look as follows:

Back to top

## Debug mode

Users can enter debug mode via the Help menu (Help->Debug). *Note: recommend only for experienced Matlab users!*

Back to top
