# Supplementary material for: Additive effects on the energy barrier for synaptic vesicle fusion cause supralinear effects on the vesicle fusion rate
Source: eLife. 2015 Apr 14;4:e05531. doi: 10.7554/eLife.05531 (PMC4426983; doi:10.7554/eLife.05531)
Supplement: Source code 1. — Custom software to analyze HS-induced postsynaptic currents written in MATLAB (only compatible with MATLAB R2013 or older). Instructions for how to use the program are in the readme file. Use on a Mac or Linux system requires specification of the location of the poi_library when asked for by the program. DOI: http://dx.doi.org/10.7554/eLife.05531.031 [file elife05531s008.zip › log_sucrose.log]

27-Feb-2015 08:24:45: Added file 2014\_10\_31\_cell04\_0001.abf in group 250mM
27-Feb-2015 08:24:45: Added 1 files to the data structure.
27-Feb-2015 08:25:37: Added file 2014\_10\_31\_cell04\_0001.abf in group 250mM
27-Feb-2015 08:25:37: Added 1 files to the data structure.
27-Feb-2015 08:25:56: Added file 2014\_10\_31\_cell04\_0002.abf in group 500mM
27-Feb-2015 08:25:56: Added 1 files to the data structure.
27-Feb-2015 08:27:07: Added file 2014\_10\_31\_cell04\_0001.abf in group 250mM
27-Feb-2015 08:27:07: Added 1 files to the data structure.
27-Feb-2015 08:27:23: Added file 2014\_10\_31\_cell04\_0002.abf in group 500mM
27-Feb-2015 08:27:23: Added 1 files to the data structure.
27-Feb-2015 08:28:46: Started fitting all data.
27-Feb-2015 08:28:46: Started fitting data.
27-Feb-2015 08:28:46: fitting block 1 of file 2014\_10\_31\_cell04\_0002.abf
27-Feb-2015 08:30:17: Started fitting all data.
27-Feb-2015 08:30:17: Started fitting data.
27-Feb-2015 08:30:17: fitting block 1 of file 2014\_10\_31\_cell04\_0002.abf
27-Feb-2015 08:32:29: Started fitting all data.
27-Feb-2015 08:32:29: Started fitting data.
27-Feb-2015 08:32:29: fitting block 1 of file 2014\_10\_31\_cell04\_0002.abf
27-Feb-2015 08:34:43: Started fitting all data.
27-Feb-2015 08:34:43: Started fitting data.
27-Feb-2015 08:34:43: fitting block 1 of file 2014\_10\_31\_cell04\_0002.abf
27-Feb-2015 08:38:37: Added file 2014\_10\_31\_cell04\_0001.abf in group 250mM
27-Feb-2015 08:38:37: Added 1 files to the data structure.
27-Feb-2015 08:38:50: Added file 2014\_10\_31\_cell04\_0002.abf in group 500mM
27-Feb-2015 08:38:50: Added 1 files to the data structure.
27-Feb-2015 08:39:10: Started fitting all data.
27-Feb-2015 08:39:10: Started fitting data.
27-Feb-2015 08:39:10: fitting block 1 of file 2014\_10\_31\_cell04\_0002.abf
27-Feb-2015 08:45:43: New parameters were stored.
27-Feb-2015 08:45:43: Finished fitting data.
27-Feb-2015 08:45:43: Finished fitting all data in 6 minutes.
27-Feb-2015 08:53:53: Failed to save the current data to a session file.
27-Feb-2015 08:57:02: Started fitting all data.
27-Feb-2015 08:57:03: Started fitting data.
27-Feb-2015 08:57:03: fitting block 1 of file 2014\_10\_31\_cell04\_0002.abf
27-Feb-2015 08:59:06: Started fitting all data.
27-Feb-2015 08:59:07: Started fitting data.
27-Feb-2015 08:59:07: fitting block 1 of file 2014\_10\_31\_cell04\_0002.abf
27-Feb-2015 09:01:53: line 120 in file 'legend'
27-Feb-2015 09:01:53: Debug sucrose\_GUI\_v2.m @line 1744: 
27-Feb-2015 09:03:03: Started fitting all data.
27-Feb-2015 09:03:03: Started fitting data.
27-Feb-2015 09:03:03: fitting block 1 of file 130521\_WT\_c01\_0003.abf
27-Feb-2015 11:34:28: Started fitting all data.
27-Feb-2015 11:34:29: Started fitting data.
27-Feb-2015 11:34:29: fitting block 1 of file 130521\_WT\_c01\_0003.abf
27-Feb-2015 11:40:06: Started fitting all data.
27-Feb-2015 11:40:06: Started fitting data.
27-Feb-2015 11:40:06: fitting block 1 of file 130521\_WT\_c01\_0003.abf
27-Feb-2015 11:46:38: New parameters were stored.
27-Feb-2015 11:46:38: line 2368 in file 'sucrose\_GUI\_v2'
27-Feb-2015 11:46:38: Debug sucrose\_GUI\_v2.m @line 2405: 
27-Feb-2015 11:46:38: Finished fitting data.
27-Feb-2015 11:46:38: Started fitting data.
27-Feb-2015 11:46:38: fitting block 1 of file 130521\_WT\_c02\_0003.abf
27-Feb-2015 11:51:05: New parameters were stored.
27-Feb-2015 11:51:05: line 2368 in file 'sucrose\_GUI\_v2'
27-Feb-2015 11:51:05: Debug sucrose\_GUI\_v2.m @line 2405: 
27-Feb-2015 11:51:05: Finished fitting data.
27-Feb-2015 11:51:05: Started fitting data.
27-Feb-2015 11:51:05: fitting block 1 of file 130522\_wt\_c09\_0005.abf
27-Feb-2015 11:55:04: New parameters were stored.
27-Feb-2015 11:55:04: line 2368 in file 'sucrose\_GUI\_v2'
27-Feb-2015 11:55:04: Debug sucrose\_GUI\_v2.m @line 2405: 
27-Feb-2015 11:55:04: Finished fitting data.
27-Feb-2015 11:55:04: Finished fitting all data in 14 minutes.
