# Supplementary figures and images for: Additive effects on the energy barrier for synaptic vesicle fusion cause supralinear effects on the vesicle fusion rate
Source: eLife. 2015 Apr 14;4:e05531. doi: 10.7554/eLife.05531 (PMC4426983; doi:10.7554/eLife.05531)

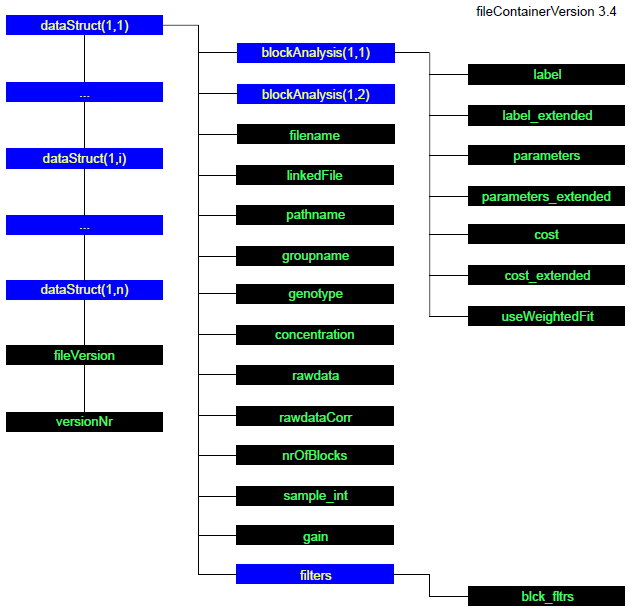

Supplement: Source code 1. — Custom software to analyze HS-induced postsynaptic currents written in MATLAB (only compatible with MATLAB R2013 or older). Instructions for how to use the program are in the readme file. Use on a Mac or Linux system requires specification of the location of the poi_library when asked for by the program. DOI: http://dx.doi.org/10.7554/eLife.05531.031 [file elife05531s008.zip › doc/dataStruct.png]

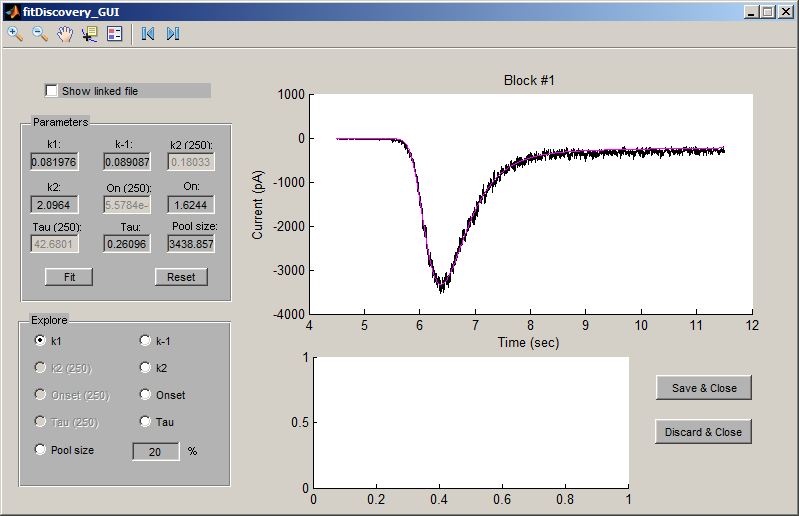

Supplement: Source code 1. — Custom software to analyze HS-induced postsynaptic currents written in MATLAB (only compatible with MATLAB R2013 or older). Instructions for how to use the program are in the readme file. Use on a Mac or Linux system requires specification of the location of the poi_library when asked for by the program. DOI: http://dx.doi.org/10.7554/eLife.05531.031 [file elife05531s008.zip › doc/exploreGUI.png]

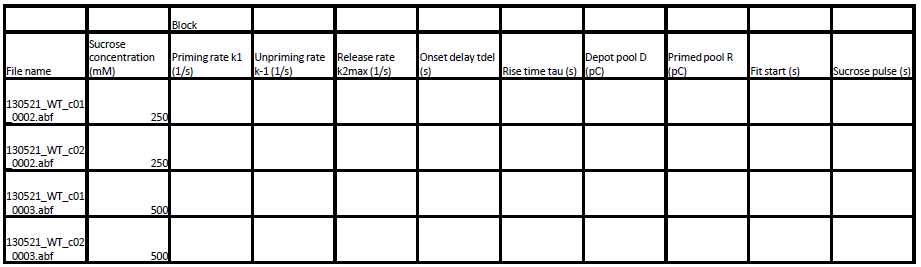

Supplement: Source code 1. — Custom software to analyze HS-induced postsynaptic currents written in MATLAB (only compatible with MATLAB R2013 or older). Instructions for how to use the program are in the readme file. Use on a Mac or Linux system requires specification of the location of the poi_library when asked for by the program. DOI: http://dx.doi.org/10.7554/eLife.05531.031 [file elife05531s008.zip › doc/exportTable.png]

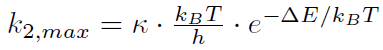

Supplement: Source code 1. — Custom software to analyze HS-induced postsynaptic currents written in MATLAB (only compatible with MATLAB R2013 or older). Instructions for how to use the program are in the readme file. Use on a Mac or Linux system requires specification of the location of the poi_library when asked for by the program. DOI: http://dx.doi.org/10.7554/eLife.05531.031 [file elife05531s008.zip › doc/eyringEquation.png]

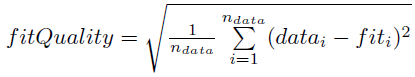

Supplement: Source code 1. — Custom software to analyze HS-induced postsynaptic currents written in MATLAB (only compatible with MATLAB R2013 or older). Instructions for how to use the program are in the readme file. Use on a Mac or Linux system requires specification of the location of the poi_library when asked for by the program. DOI: http://dx.doi.org/10.7554/eLife.05531.031 [file elife05531s008.zip › doc/fitQuality.png]

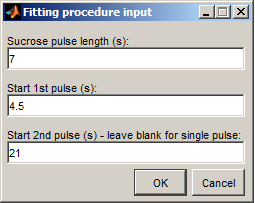

Supplement: Source code 1. — Custom software to analyze HS-induced postsynaptic currents written in MATLAB (only compatible with MATLAB R2013 or older). Instructions for how to use the program are in the readme file. Use on a Mac or Linux system requires specification of the location of the poi_library when asked for by the program. DOI: http://dx.doi.org/10.7554/eLife.05531.031 [file elife05531s008.zip › doc/fittingInput.png]

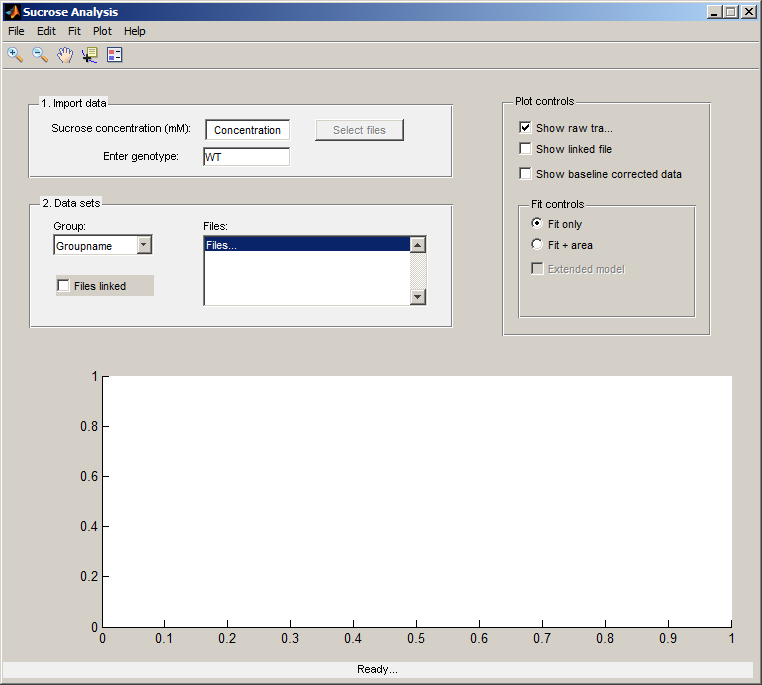

Supplement: Source code 1. — Custom software to analyze HS-induced postsynaptic currents written in MATLAB (only compatible with MATLAB R2013 or older). Instructions for how to use the program are in the readme file. Use on a Mac or Linux system requires specification of the location of the poi_library when asked for by the program. DOI: http://dx.doi.org/10.7554/eLife.05531.031 [file elife05531s008.zip › doc/initialGUI.png]

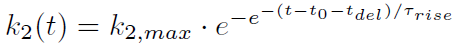

Supplement: Source code 1. — Custom software to analyze HS-induced postsynaptic currents written in MATLAB (only compatible with MATLAB R2013 or older). Instructions for how to use the program are in the readme file. Use on a Mac or Linux system requires specification of the location of the poi_library when asked for by the program. DOI: http://dx.doi.org/10.7554/eLife.05531.031 [file elife05531s008.zip › doc/k2vsTime.png]

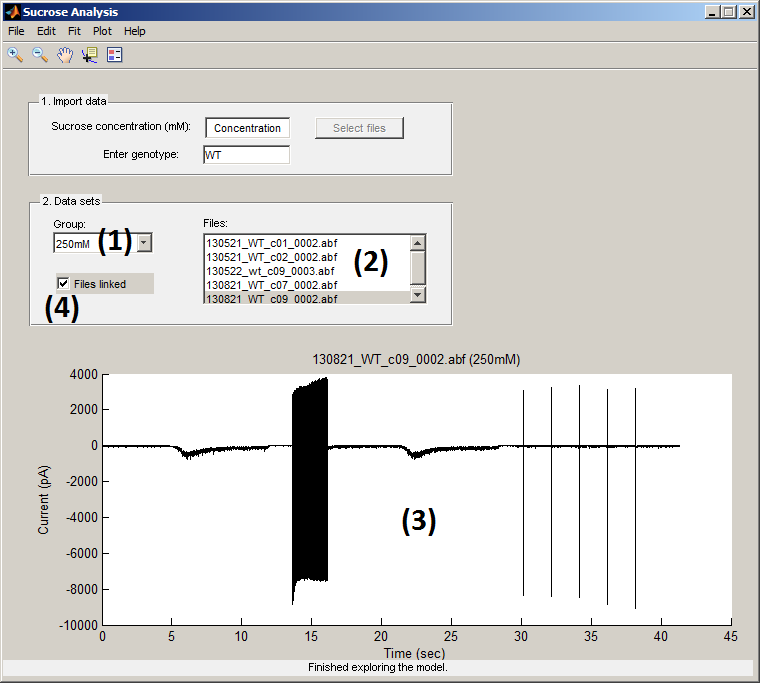

Supplement: Source code 1. — Custom software to analyze HS-induced postsynaptic currents written in MATLAB (only compatible with MATLAB R2013 or older). Instructions for how to use the program are in the readme file. Use on a Mac or Linux system requires specification of the location of the poi_library when asked for by the program. DOI: http://dx.doi.org/10.7554/eLife.05531.031 [file elife05531s008.zip › doc/loadingDoneGUI.png]

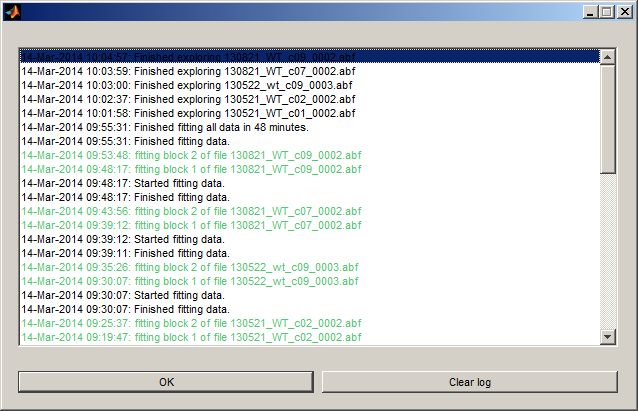

Supplement: Source code 1. — Custom software to analyze HS-induced postsynaptic currents written in MATLAB (only compatible with MATLAB R2013 or older). Instructions for how to use the program are in the readme file. Use on a Mac or Linux system requires specification of the location of the poi_library when asked for by the program. DOI: http://dx.doi.org/10.7554/eLife.05531.031 [file elife05531s008.zip › doc/log.png]

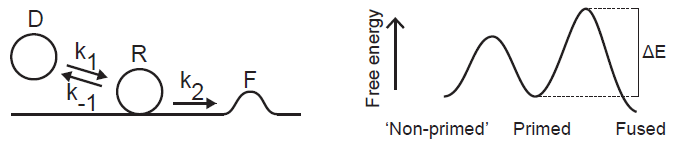

Supplement: Source code 1. — Custom software to analyze HS-induced postsynaptic currents written in MATLAB (only compatible with MATLAB R2013 or older). Instructions for how to use the program are in the readme file. Use on a Mac or Linux system requires specification of the location of the poi_library when asked for by the program. DOI: http://dx.doi.org/10.7554/eLife.05531.031 [file elife05531s008.zip › doc/model.png]

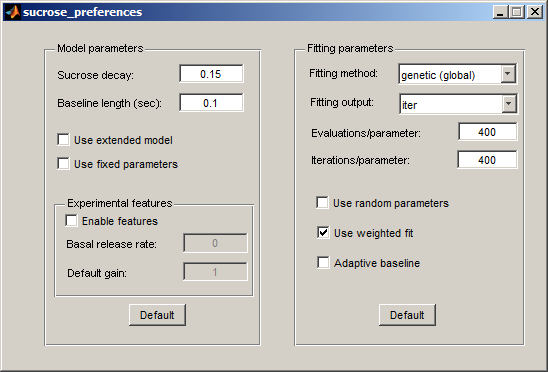

Supplement: Source code 1. — Custom software to analyze HS-induced postsynaptic currents written in MATLAB (only compatible with MATLAB R2013 or older). Instructions for how to use the program are in the readme file. Use on a Mac or Linux system requires specification of the location of the poi_library when asked for by the program. DOI: http://dx.doi.org/10.7554/eLife.05531.031 [file elife05531s008.zip › doc/preferences.png]

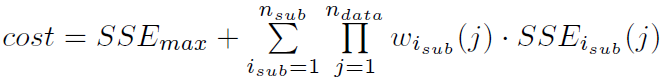

Supplement: Source code 1. — Custom software to analyze HS-induced postsynaptic currents written in MATLAB (only compatible with MATLAB R2013 or older). Instructions for how to use the program are in the readme file. Use on a Mac or Linux system requires specification of the location of the poi_library when asked for by the program. DOI: http://dx.doi.org/10.7554/eLife.05531.031 [file elife05531s008.zip › doc/weightedSSE.png]

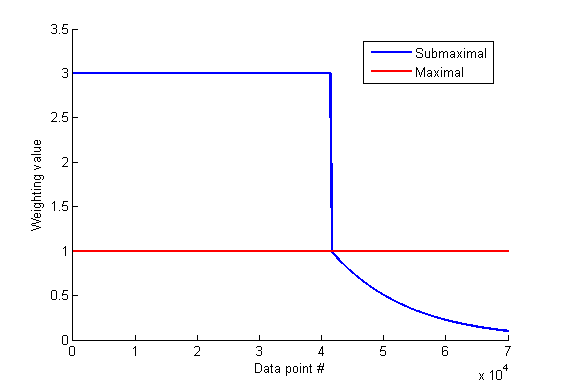

Supplement: Source code 1. — Custom software to analyze HS-induced postsynaptic currents written in MATLAB (only compatible with MATLAB R2013 or older). Instructions for how to use the program are in the readme file. Use on a Mac or Linux system requires specification of the location of the poi_library when asked for by the program. DOI: http://dx.doi.org/10.7554/eLife.05531.031 [file elife05531s008.zip › doc/weightingFunction.png]
